# The Social-Ecological System of Farmers’ Current Soil Carbon Management in Australian Grazing Lands

**DOI:** 10.1007/s00267-023-01801-4

**Published:** 2023-03-07

**Authors:** Md Nurul Amin, Lisa Lobry de Bruyn, Md Sarwar Hossain, Andrew Lawson, Brian Wilson

**Affiliations:** 1 School of Environmental and Rural Science, University of New England, Armidale, NSW Australia; 2 Department of Environmental Science, Patuakhali Science and Technology University, Patuakhali, Bangladesh; 3 Environmental Science and Sustainability, School of Interdisciplinary Studies, University of Glasgow, Dumfries, Scotland; 4 Australian Centre for Agriculture & Law, School of Law, University of New England, Armidale, NSW Australia

**Keywords:** Agri-environmental benefits, Australian grazing lands, Carbon sequestration, Market-based mechanisms, Network analysis

## Abstract

Soil carbon sequestration programmes are a way of offsetting GHG emissions, however, it requires agricultural landholders to be engaged in such initiatives for carbon offsets to occur. Farmer engagement is low in market-based programmes for soil carbon credits in Australia. We interviewed long-term practitioners (*n* = 25) of rotational grazing in high-rainfall lands of New South Wales, Australia to understand their current social-ecological system (SES) of soil carbon management (SCM). The aim was to identify those components of the SES that motivate them to manage soil carbon and also influence their potential engagement in soil carbon sequestration programmes. Utilising first-tier and second-tier concepts from Ostrom’s SES framework, the interview data were coded and identified a total of 51 features that characterised the farmers’ SES of SCM. Network analysis of farmer interview data revealed that the current SES of SCM has low connectivity among the SES features (30%). In four workshops with interviewed farmers (*n* = 2) and invited service providers (*n* = 2) the 51 features were reviewed and participants decided on the positioning and the interactions between features that were considered to influence SCM into a causal loop diagram. Post-workshop, 10 feedback loops were identified that revealed the different and common perspectives of farmers and service providers on SCM in a consolidated causal loop diagram. Defining the SES relationships for SCM can identify the challenges and needs of stakeholders, particularly farmers, which can then be addressed to achieve local, national and international objectives, such as SCM co-benefits, GHG reduction, carbon sequestration targets and SDGs.

## Introduction

Sequestering carbon in soil is a potential response to mitigating climate change (Bossio et al. 2020; Frank et al. 2017) and agricultural soil carbon management (SCM) holds much promise in this regard (Amin et al. 2016; Sykes et al. 2019; Yang et al. 2019). Farmers’ ability to sequester soil carbon will depend on various biophysical and socio-economic factors in their management environment (Bossio et al. 2020). In addition to emissions reduction goals, SCM can provide a range of co-benefits, such as improved water-holding capacity, soil fertility (Frank et al. 2017; Zomer et al. 2017) and productivity (Branca et al. 2013). Land-based SCM could reduce carbon emissions by 23.8 Gt CO_2_–equivalent per year (25% contribution to total anthropogenic emissions reduction) (Bossio et al. 2020; Lal 2004). A number of international and national initiatives highlight the importance of SCM for mitigating climate change, including the COP21 initiative to increase soil organic carbon stocks by 0.4% per year (Minasny et al. 2017; Rumpel et al. 2018) and the Australian Government’s Emission Reduction Fund (ERF) programme (Australian Government 2020; Verschuuren 2017). Co-benefits from SCM could also address several UN Sustainable Development Goals, including Zero Hunger (SDG 2), Climate Action (SDG 13) and Land Degradation Neutrality (SDG 15.3) (Amin et al. 2020; Kust et al. 2017), and help to build political, financial and technical momentum to address these goals (Vermeulen et al. 2019). However, the challenge for SCM is dealing with the contrasted knowledge of biophysical conditions and influence of SCM practices to increase SOC levels (Demenois et al. 2020). Other barriers from a farmer’s point of view include policy uncertainty (e.g., contract terms), cultural values, or soil stewardship ethics, the latter of which have been shown to influence transitioning to SCM as a climate mitigation strategy more than efficacy (Buck and Palumbo-Compton 2022).

To study the complex relationships and draw lessons for ecosystem resilience, Ostrom (2007, 2009) recognised a social-ecological system (SES) framework composed of first- and second-tier concepts that interact with each other to produce outcomes. Outside the first- and second-tier concepts were social, political and economic and ecosystem settings. The outcome of these interactions influence the system components, subsystems and other SESs. Ostrom’s SES framework organises the empirical and theoretical variables to provide sustainable solutions to problems (Ostrom 2007, 2009), and enhances the sustainability of public policy by identifying particular issues from parts of the SES framework that might influence policy (Ostrom 2009). This SES framework has been used to understand the complexity of regional sustainability (Chen et al. 2022; Hossain et al. 2020b; Hossain et al. 2020a; Willcock et al. 2016) and the impact on ecosystem services (Lopes and Videira 2019; Hossain et al. 2016; Hossain et al. 2017), transformation systems and product accommodation (Marshall 2015), and fisheries and water management (de Wet and Odume 2019; Galappaththi et al. 2019). A systematic review of SCM research in Australia found a limited understanding of the type of SES components and how they interact to influence Australian farmers’ soil carbon management (Amin et al. 2020). In addition, the review also found that the analysis of SCM using a SES approach had yet to be undertaken internationally (Amin et al. 2020). The majority of scientific studies, in Australia, emphasised soil carbon accumulation processes and ecological triggers or settings for soil carbon improvement, with little consideration of farmers’ perspectives or identification of the influential socio-ecological system components in SCM (Amin et al. 2020; Gosnell et al. 2020). Due to scant research in this area, understanding the SES components of SCM and their relationships is limited.

It is important to understand the potential for offsetting GHGs through sequestering carbon in grazing lands (de Otálora et al. 2021; Reich et al. 2020; Rey et al. 2017) because of the prevalence of cattle and sheep grazing enterprises in Australia (around 336 million hectares or 50% of land area) and the opportunity it presents (Climate Work Australia 2021). Given the favourable climatic conditions, lower land clearance and dominance of grazing agricultural practices, the study area is assumed to have medium to high potential for achieving the benefits of SCM, namely offsetting GHG emissions and restoring soil health (Waters et al. 2020; Wilson and Lonergan 2013). We interviewed long-term practitioners (*n* = 25) of rotational grazing in high-rainfall lands of New South Wales, Australia to understand their current social-ecological system (SES) of soil carbon management (SCM). By studying long-term practitioners of SCM in grazing lands, it will identify those social and ecological system components or features that influence their current SCM and where soil carbon policy initiatives, market-based mechanisms or individual behaviour change can affect the potential engagement of other farmers with less experience in SCM. The aim was to identify those components of the SES that motivate them to manage soil carbon sequestration and also influence their potential engagement in SCM programmes. Utilising first-tier and second-tier concepts from Ostrom’s SES framework, the interview data were coded to identify the features (i.e., second-tier variables) that characterised the farmers’ SES of SCM (Supplementary Information Fig. 1). Our study focused on the following research questions:

1. What are the current social and ecological features that influence SCM at the farm level?
2. How do these features interact to influence farmers’ SCM?
3. What type of feedback loops operate among these features?
4. What are the implications of the current SES of SCM for farmers and policymakers?

‘Methodology’ introduces the study area, methodological approach for examining the connectivity and interrelationships of the SES features in the SES of SCM. ‘Results’ 3 discusses the connectivity between the SES features of SCM (RQ-1, ‘Social-ecological system network diagram of study farms’), stakeholders’ perspectives of social-ecological relationships (RQ-2, ‘Farmer and service provider perspectives of SES relationships for SCM’), the interaction and feedback in the SES for SCM (RQ-3, ‘Feedback loops in SES for SCM’) and implications of the current SES of SCM for farmers and policymakers (RQ-4, ‘Discussion’).

## Methodology

### Study Area Description

**Fig. 1:**
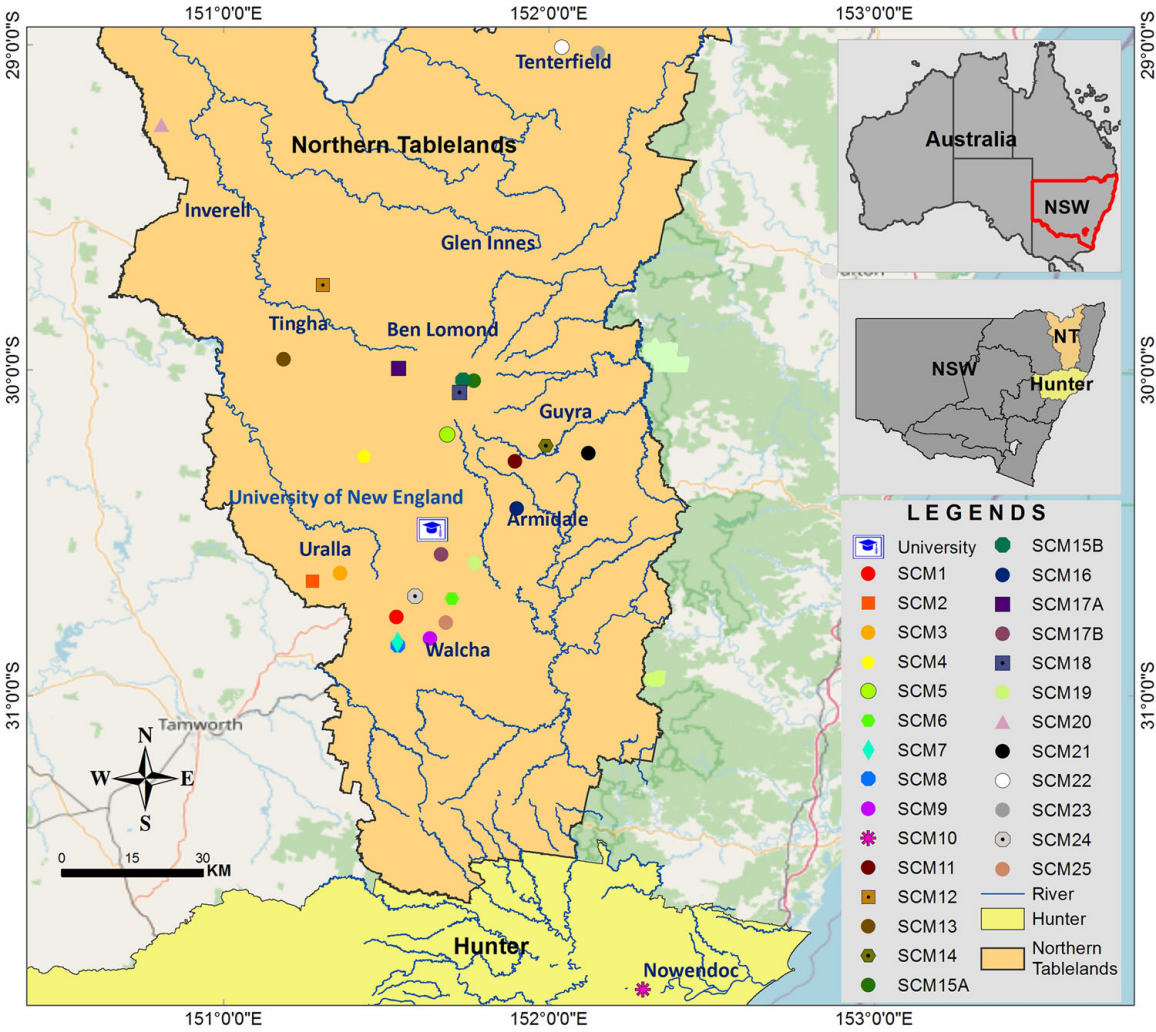
Farm locations in the study area. The abbreviation SCM followed by a number denotes anonymous farm identities, and a letter after a number indicates that those farms are owned by the same farmer (e.g., SCM17A, SCM17B)

The system boundary for the SCM SES is the agro-ecological region of the Northern Tablelands and into the Upper Hunter Valley, NSW, Australia, and within this boundary a purposive sample was selected for study (i.e., 25 farms), as explained in ‘Farm-level interviews’ (Fig. 1). In this area, 68% of land is occupied by agriculture (2.11 million ha), with an estimated agricultural commodity value of $217.8 million (Alford et al. 2003). The participating farms were sheep and cattle enterprises, which are the dominant agri-enterprises, contributing 86% of the total value for the study area. The region produces wool fibre and meat (beef and lamb) at 42 and 44%, respectively (Alford et al. 2003). Farms in the region, including those studied, are predominately perennial native pastures or a combination of native and introduced perennial pastures with high ground cover throughout the year (Alford et al. 2003). Farms in the study area are located across a broad plateau that ranges in altitude from 750 to 1200 metres above sea level and is in a temperate climate zone where more than 60% of the annual rain falls over summer, which is the peak plant-growing season. Maximum temperatures usually remain below 30 °C and annual mean minimum temperatures are around 7 °C (Wilson and Lonergan 2013). Average annual rainfall is 750–800 mm with frequent seasonal droughts (1:3.5 years) and less frequent serious droughts (1:10 years) (Alford et al. 2003; Wilson and Lonergan 2013). Studied farms are either located on low-fertility soils (granite and sedimentary geology; Chromosol), or comparatively fertile soil (basalt geology; Ferrosol and Dermosol) (Office of Environment and Heritage 2018).

### Overview of Data Collection and Analysis

**Fig. 2:**
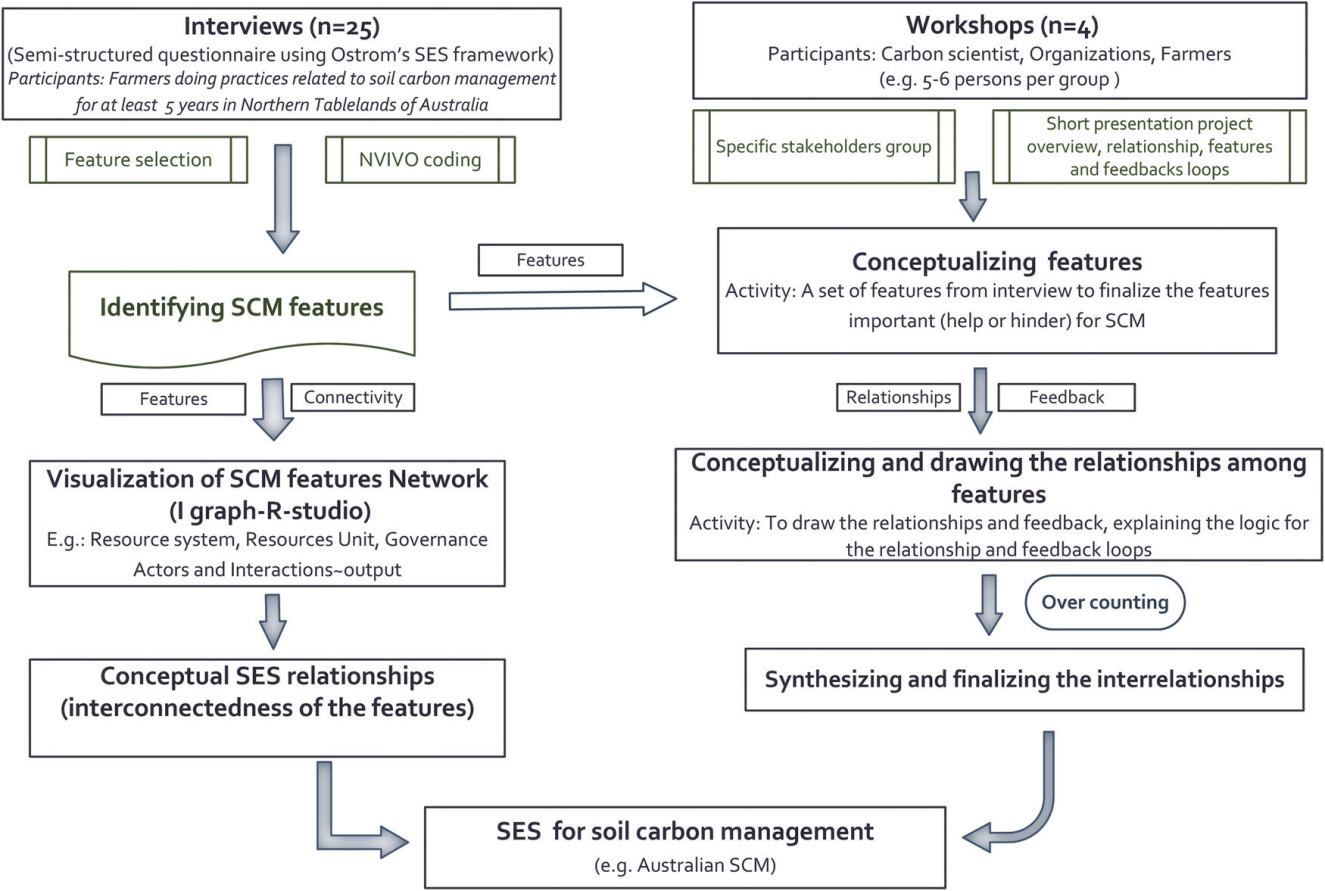
Schematic diagram of the research steps undertaken

A mix of quantitative and qualitative research methods (Fig. 2) were employed in four consecutive steps to reveal the SES of SCM for purposively selected grazing farmers in the study area, with detail on each provided in following sections: (i) Semi-structured farmer interviews for identifying the SES features of farmers’ SCM utilising first- and second-tier concepts from Ostrom’s SES framework were utilised to design the interview questions; (ii) Network Analysis (NA) on farmer interview data was used to analyse the connectivity and importance of the SES features of farmers’ current SCM; (iii) The features identified from the farmer interview and NA were presented to farmers and service providers at a workshop to validate their use as part of a SES for SCM, which was collaboratively constructed by participants using a causal loop diagram (Haraldsson 2004). The workshop was also used to then identify the types of interactions and feedback loops (post-workshop) based on causal loop diagram; and (iv) finally a consolidated SES for SCM that integrated the farmer and service-provider workshop outcomes was developed using a causal loop diagram. Human Research Ethics Clearance for all the research data collection and procedures was approved by the University of New England, Australia (Approval No. HE19-149).

#### Use of Ostrom’s social-ecological system (SES) framework in interview design

Ostrom argued that social-ecological relationships are complex and can only be understood by examining the whole system as an interconnected set of features (Rocha et al. 2020). To structure the interview questions, we utilised Ostrom’s (2007, 2009) first-tier concepts (Supplementary Information Fig. 1) i.e., resource system, resource unit, governance, actors, and interaction-output and those second-tier concepts applicable to a grazing system. The first-tier concepts of an SES framework are the resource system (e.g., water system, wildlife), the resource unit (e.g., the types of animals or amount of water flow), the governance system (e.g., the organisation or authority that manages the resource systems or the mode of management), the actors or users (e.g., the individuals who use the resource system) and interactions-outcomes (e.g., efficiency, sustainability - titled as ‘interaction-output’ in this study) (McGinnis and Ostrom 2014; Ostrom 2007, 2009). In this study, interaction-output consist of changes in soil parameters, pasture and animal productivity and well-being of the practitioners. Ostrom’s SES framework is an approach to understanding the complex characteristics of a system and the relationships (e.g., interactions and feedback) between the SES features which are unique compared to existing system -based frameworks.

#### Farm-level interviews

The semi-structured interview schedule design enabled a structure that would allow sub-themes to emerge, but was framed around accepted SES first-tier concepts of Ostrom’s SES framework (SI – Semi-structured interview). The interview schedule was pre-tested with several long-term practitioners of rotational grazing, and with an agricultural consultant from the study region. SCM practices identified by Dumbrell et al. (2016), at a national–level, were included in the interview schedule, despite some of the practices being irrelevant to the farmers being interviewed. Interviewees were purposively selected with the assistance of two intermediaries (Local Land Services, a NSW State government natural resource management agency, and Southern New England Landcare, a local non-government ‘Landcare’ group). Inclusion criteria were beef and sheep grazing farmers who had at least five years’ experience implementing at least two types of SCM practices as identified by Dumbrell et al. (2016) (Box 1), chosen because they were highly experienced in grazing management at farm-level, and were operating under similar environmental conditions. Semi-structured interviews with case study farmers (*n* = 25) were conducted between November 2019 and February 2020. Full description of farmer and farm (years of experience, farm size, and SCM practices) are available in Amin et al. (2023) and Supplementary Information Table 2.

The structure and organisation of the interview schedule was based on Ostrom (2009), and responses of the farmers to the interview questions were used for the identification and placement of SES features of SCM. The interview questions were open-ended and designed to draw out the SES features of SCM organised under each of Ostrom’s first-tier concepts (resource system, resource units, actors, governance systems and interaction-output) (Supplementary Information Fig. 1) (McGinnis and Ostrom 2014; Ostrom 2007, 2009). Hence deductive reasoning for placement under the first-tier, with inductive reasoning for placement of new second-tier variables or features. The questions canvassed three aspects of the farming operations: (1) demographics (age, gender, education, farming experience, farm debt status, soil types, farm type and proprietorship); (2) farm features and their interrelationships; and (3) directional soil responses (soil pH, soil moisture, soil structure and nutrients) to SCM practices (increase, decrease). The interviews lasted up to 90 min and were recorded, with their consent and transcribed. Data was coded from the relevant part of the interview using NVivo 12 Plus to sub-themes (e.g., Supplementary Information Table 8) based on Ostrom’s definitions of first and second-tier concepts, but also allowing for new sub-themes or features to emerge under one of the relevant five first-tier concepts (resource system, resource units, actors, governance systems and interaction-output) (McGinnis and Ostrom 2014; Ostrom 2007, 2009). This process of coding data from the interviews was over multiple readings of the transcript. Final lists of SES features for SCM were drawn from the detailed farmers’ interviews and were the basis for discussion at the farmer and service provider workshops (‘Stakeholders’ workshop’).

##### Box 1 Soil carbon management practices (Dumbrell et al. 2016)

No-till cropping practices

Bio-char application

Mulching on bare soil

Increase area for pasture by decreasing area for crop

Inter cropping with perennial pasture

Perennial pasture planting

Tree belt planting

Rotational grazing implementation

Stubble retention after crop harvest

Legume in pasture

Others (specified by farmers e.g., Grazing management)

#### Network analysis

Network analysis was used to identify the current connectivity and influence of the SES features of SCM. One-mode networks (defined in Box 2) were used to visualise the connections of second-tier variables or features on each of the 25 farms. Moreover, one-mode networks visualises the importance of a second-tier variables or feature in the network based on the size of the circle and the number of connections to other second-tier variables or features. Two-mode networks (see Box 2) show visually how the SCM second-tier variables or features are connected to each other in the network and how farms are connected to each other on the basis of these SCM second-tier variables or features (Supplementary Information Table 9).

In this study, a one-mode network was used to visualise the connections among Ostrom’s five first-tier concepts and underlying SES features of SCM. A numeric distribution network was produced from the farmers’ interview data on the features they individually nominated to influence SCM in the study area, and combined to form the network (e.g., Supplementary Information Table 1). We studied specific responses from all interviewed farmers individually on each feature they identified, and each feature was given a weighted number (‘1’ or ‘0’) depending on the response. To describe the influence of the ‘interaction-output’ features on other SES features within the network, relationships ‘with influence’ were given a value of ‘1’ and ‘no influence’ were given a value of ‘0’ (Supplementary Information Table 1). For example, when an interviewed farmer responded that an SCM practice has increased because of a feature’s influence, that outcome was assigned as ‘with influence’ and given a value of ‘1’. Also, the farmers’ responses ‘with influence’ to the question of production optimisation from using an SCM were assigned as ‘yes’ and given a value of ‘1’ (Supplementary Information Table 1).

Features with multiple response options (e.g., size of the farm, number of farms under farming, farming type, proprietorship, loan status) were used to examine the numeric relationship of the response with the outcome and resource unit features in the network (e.g., number of small farms that agreed to ‘improvements in nutrient cycling were due to SCM practices and/or improvement in soil health’) (Supplementary Information Table 1).

In the completed network diagram for this study, each of the SES features of SCM are represented as circles (e.g., soil health, production potential). The lines that connect one feature to another feature are referred to as ‘links’ (Box 2). The width of the lines indicates the number of the links (e.g., number of farm responses on each connection). In the one-mode network thicker lines between features indicates more links (e.g., SCM practices increase soil erosion control) and a thinner lines indicates fewer links between features (e.g., less understanding of soil biodiversity change leads to less adoption of SCM practices). For both of the networks, the number of connections to a feature represents the connectivity of that feature in the network. The resultant density of the two-mode network reveals the degree of connectivity of the SES features of SCM in the study area. We used the package ‘igraph’ in RStudio version 1.1.456 to analyse network properties and visualise the network diagrams, broadly following Rocha et al. (2015 and 2020) and Ognyanova (2016).

##### Box 2 Glossary of terms

Soil carbon management (SCM): Practices and techniques that accumulate carbon in the soil, e.g., nutrient optimisation, reduced soil disturbance, restoration of grassland biodiversity and grazing management

Social-ecological systems (SES): Systems that emerge from human interactions with their environment, comprising a multi-level interconnected set of features shaped by each other.

Co-benefits: Contemporaneous societal, environmental and private benefits of adopting soil carbon management, in addition to GHG sequestration, e.g., increased soil moisture, soil biological activity, mental health, and shelter for livestock.

Feedback loops: Reciprocal relationships of system features that either balance (i.e., moderate) or reinforce the effects of features.

Reinforcing feedback loops: Those feedback loops that reinforce, accentuate, or magnify the initial change in the systems, e.g., sustainable land management (SLM) practices reinforce increased farm productivity and flexible financing supports management practices then greater adoption of SLM could ensure more production.

Balancing feedback loops: Those feedback loops that balance, moderate or oppose the initial change in the system, e.g., drought reduces soil moisture and grass production, but by incorporating mulch onto bare soil greater soil moisture is retained, which can counterbalance the effect of low rainfall and ensure farm production.

Second tier variables or Features and links: Each feature (e.g., farmer, soil type) is a circle in the network diagram and lines from one feature to another are links.

One-mode network: A network where the set of features and linkages are similar to each other. A one-mode network was used in this study to visualise the connections among the 51 features in the study area on each of the 25 farms.

Two-mode network: A network where there are two different sets of features, and links exist only between features in different sets. In this study, a two-mode network was used to visualise (1) How the SCM features were connected to each other, and (2) How farms were connected to each other on the basis of the SCM features.

Interactions: Influences of any feature on another feature. Interactions that increase or intensify other features are positive interactions and interactions that reduce other features are negative interactions.

Density of relationships: The connectivity of the features (e.g., SCM practices) in a network (typically expressed in percentages).

Service providers: Actors who work in a private or public capacity, usually science trained, and provide information, guidance or training on soil and land management to landholders.

#### Stakeholders’ workshop

Once the features of the SCM SES were identified from the farmers’ interviews and were examined pre-workshop through NA, the retention of features in the SES, and relationships (interactions and feedback loops) between features were validated through four participatory workshops conducted with farmers (*n* = 2) and service providers (*n* = 2) (Fig. 2). Half of interviewed farmers participated in the farmer workshops. Those interviewed farmers who previously had agreed to attend a workshop were contacted from two distinct locations (Uralla-Kentucky-Walcha, and Guyra–Ebor). The participants in the service provider workshops were invited, and all science-trained to tertiary level, and had not taken part in the interviews. They were currently working in an advisory or education/training role as agronomist and/or scientist for State government organisations (e.g., Department of Primary Industry, Local Land Services that manage natural resources and land rates), non-government organisations (e.g., Southern New England Landcare, Agricultural Consultancy Company), and agricultural educator in holistic grazing management. To allow comparison between workshops, we used the same facilitator, structure, timing and list of SCM features derived from farmer interviews and NA (‘Overview of data collection and analysis’ and ‘Network analysis’).

Workshops (*n* = 4) were organised between October and December 2020 in facilities that were convenient for participants to travel to. Each workshop had four to six participants and lasted about 180 min. Participants were of mixed age (26–79 years). The workshops were repeated for both the service providers (*n* = 2) and farmers (*n* = 2) to ensure saturation of information and to minimise redundancy. Separate workshops for farmers and service providers ensured that the perspectives of each group did not influence the other group, and participants would feel unencumbered to voice their opinion.

At the workshops, we presented the list of SCM features that were the product of the coding of all farmer interviews, to participants, who reviewed the list individually. Participants were asked to retain features that, in their experience, help or hinder their current ability to manage soil carbon at farm level and to discard features that neither helped nor hindered. A final list of retained features was reached through group discussion and consensus agreement by all participants as to why a feature should be retained. Any disagreement was noted and considered when all four causal loop diagrams from the workshops (Supplementary Information Figs. 4–7) were consolidated. The participants first attached the retained features onto a white board. The participants at the workshop placed the features closest to SCM that were most important and those features that were further away from SCM were less important. The participants were then asked to decide the type of linear interaction between features if it was positive and/or negative. Post-workshop the type of interactions were determined by researcher if the feedback loop (Sterman 2000; Ford 2010) was a positive or negative one (i.e., reinforcing or balancing feedback loop) (Box 2).

We compared the resulting SES for SCM from each workshop for similarities, differences and redundancies. Finally, the SCM interactions were consolidated and visualised using the system dynamic (SD) modelling platform STELLA version 1.8.2.

## Results

The interviewed farmers were all undertaking two to three SCM practices, with the average length of experience at 26 years (Supplementary Information Table 2). More than 50% of the total studied farms were dominated by perennial native pastures. Most farms (55%) are located on low-fertility soils and the remainder (45%) are comparatively fertile soil, based on parent material. The farmers interviewed were of mixed age (40–79 years), managing predominantly grazing enterprises, with 70% of farms being more than 500 ha and commercially operating (Supplementary Information Table 2). In the process of qualitatively coding of the farmer interviews a total of 51 SCM features in the studied grazing regimes with variable influence identified through Network Analysis were identified (Table 1, Supplementary Information Table 3).

**Table 1:** Soil carbon management (SCM) features based on farmer interviews

First-tier concept of SES	Features or Second-tier variables
Resource System (RS)	Geographical location, size of the farm, number of farms, farm type (e.g., grazing), Proprietorship (e.g., family farm), Loan status, Soil type (e.g., fertile/non-fertile), Soil health
Resource Unit (RU)	Production potential, SCM practices, climate, change of income, agri-environmental benefits, SCM cost
Governance (G)	Support of government organisations, support of non-governmental organisations, own farm research grant, scientific support (e.g., soil test support), government investments, private investments, carbon pricing and monitoring, certainty of payment, training and education support, expert information, soil carbon policy, social network, trusted expert network
Actors (A)	Government officer, independent advisors, farmers, scientists, education institute, soil stewardship ethics, SCM attitude, technologies available, trust
Interaction-output (I-O)	pH level, soil moisture, soil structure, soil biodiversity, landscape aesthetics, soil water-holding capacity, soil erosion, soil nutrients, soil carbon content, mental health, shelter for livestock

### Social-Ecological System Network Diagram of Study Farms

**Fig. 3:**
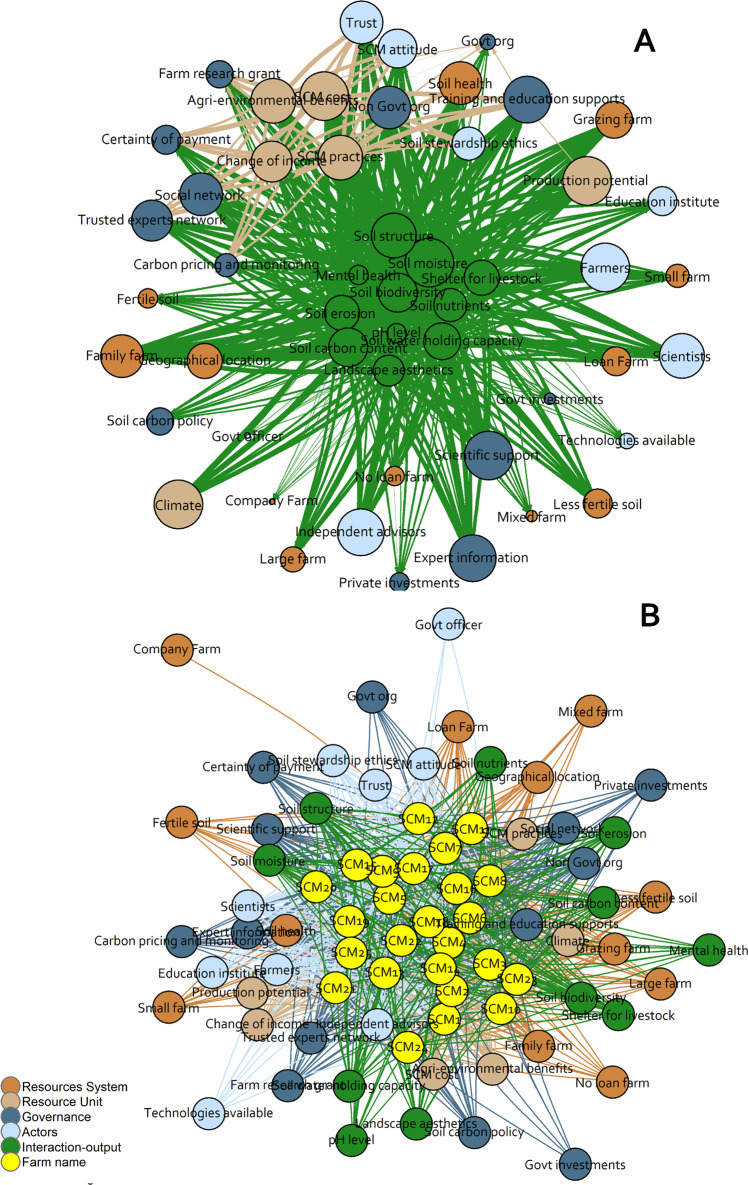
SCM features connectivity network: (**A**) One-mode network of SCM features demonstrating the connectivity and importance of features in the SES system. **B** Two-mode network of SCM features demonstrating closeness of features to the cluster of farms. Frequency of lines represents connectivity between features (Source: Supplementary Information Table 3, Supplementary Information Table 9)

The network diagrams (Fig. 3) present features as circles (*n* = 51) (Table 1, Supplementary Information Table 3) and interactions as lines between features (*n* = 483). High frequency of lines occurs where a high proportion of farmers have connections from one feature to many other features, and high density of lines occurs where a high proportion of farmers have connections between two specific features. The one-mode network analysis is shown in Fig. 3A. The size of the circle indicates the importance of the feature to the network. The most important features (with the larger circles) in each of the five first-tier concepts were optimised production potential, SCM practice (resource unit), soil health (resource system), independent advisors, trust, farmers (actors), training and education support, social network, scientific support, non-government organisation (governance), soil moisture and soil structure (interaction-output) (Fig. 3A, Supplementary Information Table 3). Overall, the most important set of features (with the greatest number of connections) were within the interaction-output first-tier i.e., soil moisture and soil structure. The important features within the first-tier resource unit were SCM practices, SCM cost and agri-environmental benefits. SCM practices was a prominently connected feature in the network (23 out of 25 farms), and was highly influenced by - SCM outcomes (e.g., improved soil health and soil moisture) and vice versa. The network importance of other resource system features such as proprietorship (e.g., family or company farm), debt status (in-debt or debt-free farm), soil type (high or low fertility) and farm type (e.g., grazing) could not be identified. Geographical location (resource system) had a weak connection to a farmer’s SCM as this relationship was predetermined especially if the farm was a family-operated one (Fig. 3A). Climate was a less densely connected feature (15 out of 25 farms); however, connectivity (number of connections) and importance (circle size) of climate in the network was high (Fig. 3A). Farmers were intrinsically motivated to undertake SCM irrespective of climate conditions; however, they recognised the importance of climate for soil carbon storage.

The two-mode network shown in Fig. 3B shows the closeness of features (76 circles and 828 links) to the case study farms (*n* = 25). In this network, the most densely connected features to the case study farms (*n* = 25) were SCM practices (*n* = 23 links), SCM cost (*n* = 21 links), climate (*n* = 15 links), training and education support (*n* = 15 links), and other farmers who were in close proximity to the farms (*n* = 15 links). Farmers largely accept that they cannot control climate, and for the region it is relatively uniform thus the majority are exposed to the same weather and climatic risks, and the consequences for SCM and storage, i.e., if there is long periods of drought and low production of biomass. Other closely connected features in the network were soil moisture, soil structure, soil health and farm production potential. The features with fewest connections to farms were government organisations, government support and technology availability. Governance features of soil carbon policy, carbon pricing and monitoring, government organisations, investment and support were distantly connected to the farms (Fig. 3B). Overall, in the two-mode network, the density of relationships between all features was low (30%), which suggests the SCM network in the study area has potential for improvement (Fig. 3B).

### Farmer and Service Provider Perspectives of SES Relationships for SCM

**Fig. 4:**
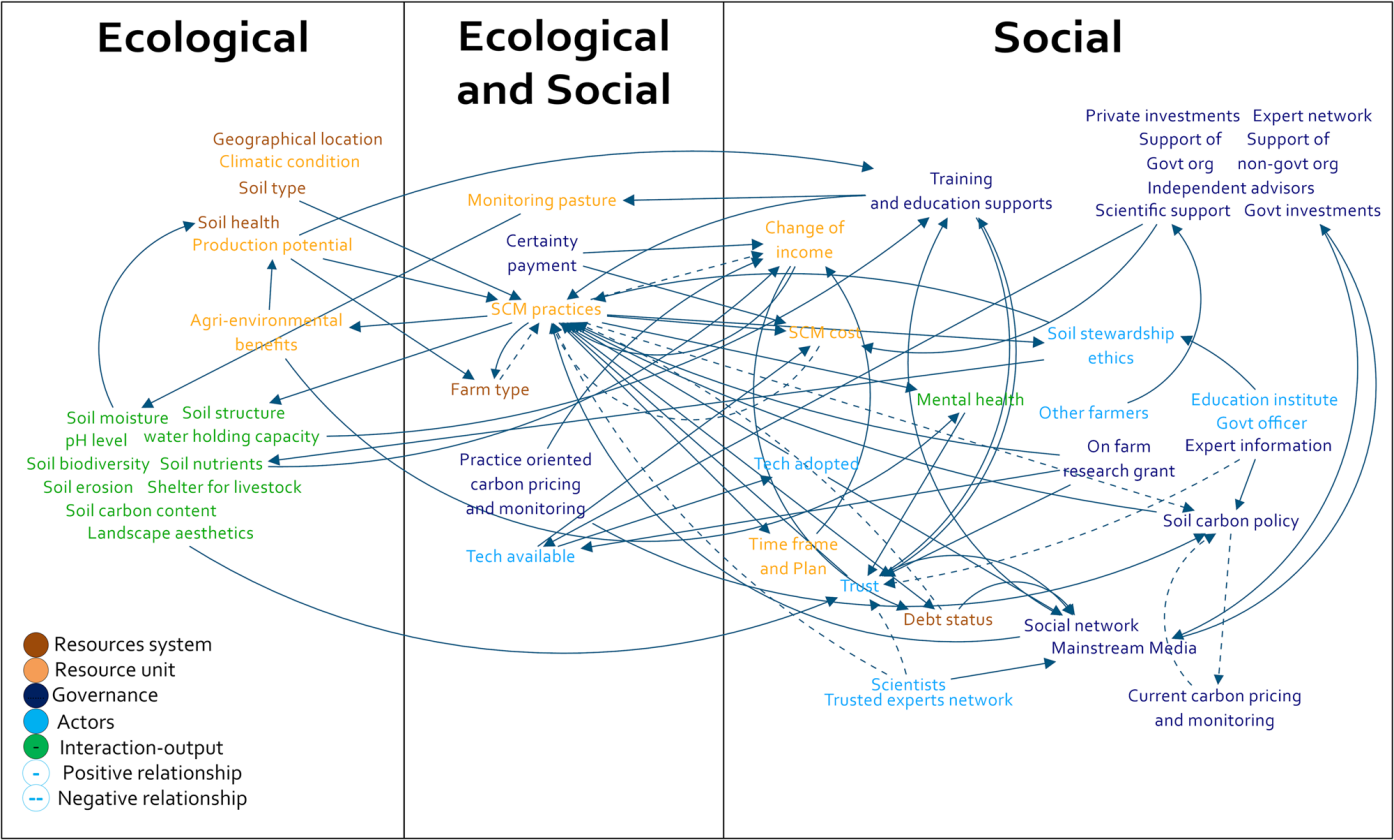
Consolidated social-ecological system (SES) for soil carbon management (SCM) in high rainfall grazing lands. Interactions were positive (solid line) and negative (dashed line) between the identified SCM features, and features were categorised under Ostrom’s SES first-tier concepts (Ostrom 2009)

Supplementary Information Tables 4–7 summarise the interactions between SES features that participants in the farmer and service-provider workshops identified as positive, negative or mixed for SCM (see also Supplementary Information Figs. 2 and 3 for farmers and Figs. 4 and 5 for service providers). Notably, for farmers, the most influential features (with all positive interactions) of the SES were the co-benefits of SCM, trust, a soil stewardship ethic, training and educational opportunities and their social networks.

Co-benefits encompassed a wide range of features from agronomic factors to mental health and landscape aesthetics. Both farmer workshops highlighted that the accrual of co-benefits from SCM practices positively influenced other features such as production potential, soil health and support of other farmers for SCM (Supplementary Information Figs. 2 and 3). Supplementary Information Figs. 2 and 3 show that farmers believe that the agri-environmental benefits of SCM practices positively influence the production potential of the farm and improve soil health. Co-benefits of SCM practices (e.g., improved soil moisture, nutrients, water-holding capacity and soil structure) positively influence interest in training and educational support. The farmers’ social network was considered to have mixed influence (negative or positive) on the existing SCM practices (Supplementary Information Fig. 2). While social networks were considered mostly positive, they could also have negative effects where peer pressure undermined innovation in management. Supplementary Information Fig. 2 shows that a high level of soil stewardship positively influences SCM practices and thereby enhances the co-benefits of SCM. Farmers’ interest in SCM was supported where there was trust between actors and a strong soil stewardship ethic was present, which they believed could, in turn improve income to further invest in SCM practices (Supplementary Information Fig. 2).

Soil carbon policy was either positively or negatively influenced by carbon pricing and monitoring. The farmer workshops tended to elicit negative views of current soil carbon policy, pricing and monitoring mechanisms, and a lack of technology for taking advantage of policies and soil carbon pricing was noted. Service providers also emphasised the positive effects of a soil stewardship ethic but tended to give more weight than farmers to features such as external investment (public and private), carbon policy and the role of extension specialists. Service providers were also circumspect about carbon pricing and monitoring mechanisms but gave qualified support for their potential impact on farmers’ income streams. In the absence of clear pricing arrangements, service providers were generally not convinced that SCM practices would result in a change of income. In addition, future investment by government and private providers for on-farm research would positively influence the testability of the required technology and its adoption, which ultimately influence SCM (Supplementary Information Fig. 5).

## Feedback Loops in SES for SCM

The workshop participants identified a number of interactions that can be summarised by six reinforcing and four balancing feedback loops. Service providers identified a balancing feedback loop involving debt status (loop 1 A, Table 2 and Fig. 4); that is, those farmers with higher farm debt might be less likely to take up SCM practices. However, if the outcomes of soil carbon management such as higher perennial cover, improved soil health and agri-environmental benefits led to positive mental health and other benefits then farmers might be likely to continue with SCM producing a balancing feedback loop, service providers suggested, compensating for higher debt levels (loop 1 A, Table 2 and Fig. 4). For farmers, debt status was not part of their consideration when undertaking SCM practices and it was a reinforcing feedback loop (loop 1B, Table 2, Fig. 4), as the focus was on the outcomes of SCM practices, such as improved mental health and agri-environmental benefits that was more influential in leading to ongoing commitment to SCM practices. A positive reinforcing feedback loop was found between SCM co-benefits, soil health and production potential (loops 2 and 7, Table 2 and Fig. 4). SCM co-benefits were considered to lead to wider soil health improvement and increased production potential of the farm, and were therefore likely to increase farm production (e.g., livestock and productivity gains) which, in turn, reinforces farmers’ SCM and other co-benefits.

**Table 2:** Identified feedback loops of the social-ecological system for SCM in grazing lands post-workshop from farmer and service provider participants causal loop diagram

Loop No.	Feedback loop type	Connected SES features	Participant type
1A	Balancing (−/+)	Debt status - SCM practices - agri-environmental benefits - mental health - trust - SCM practices – Debt status	Service providers
1B	Reinforcing (+/+)	SCM practices - agri-environmental benefits - mental health - trust - SCM practices	Farmers
2	Reinforcing (+/+)	SCM co-benefits - soil health, production potential - SCM practices - SCM co-benefits	Both types
3	Balancing (−/+)	SCM cost - SCM practices - SCM co-benefits - change of income - SCM practices –SCM costs	Both types
4	Reinforcing (+/+)	SCM practices - timeframe and plan - change of income - SCM practices	Farmers
5	Reinforcing (+/+)	Social network - training and education support - SCM practices - debt status - social network	Farmers
6	Reinforcing (+/+)	Training and education support - SCM practices - SCM co-benefits - training and education support	Farmers
7	Reinforcing (+/+)	SCM practices - SCM co-benefits - production potential and soil health - SCM practices	Both types
8	Balancing (−/+)	Production potential and soil health - farming type - SCM practices - SCM co-benefits - SCM practices - production potential and soil health	Service providers
9	Reinforcing (+/+)	Social network - SCM practices - SCM co-benefits - production potential and soil health - SCM practices - social network	Farmers
10	Balancing (−/+)	SCM cost - SCM practices - SCM co-benefits - production potential and soil health - SCM practices –SCM cost	Both types

The implementation of SCM may initially increase costs but was considered to be balanced by the positive effect of the co-benefits and thus farmers were more likely to continue with SCM, and in the long-term SCM costs would be compensated by other co-benefits, thus producing a balancing feedback loop. For example, improved SCM could improve farm production and SCM costs would be compensated, even during adverse climatic conditions, by ensuring adequate ground cover, improved soil moisture retention, and sufficient nutrients to maintain the system (balancing feedback loops 3 and 10, Table 2, Fig. 4). In the case of feedback loop 4, appropriate time frames and planning for SCM increase income over the long-term and increase the co-benefits, which in turn maintain SCM practices. Farmers’ participation in existing social networks (e.g., Landcare groups, and farmers’ Facebook groups) increases interest in training and education, possibly leading to higher SCM practice uptake, improved farm production, reduced farm debt and increased farmers’ interest in participating in farmers’ social networks to seek out further information for SCM (reinforcing feedback loops 5 and 9, Table 2 and Fig. 4).

The training and education reinforcing feedback loop (loop 6, Table 2 and Fig. 4) confirms farmers’ interest in improved support for training and education from government and other potential sources. From their perspective, such support leads to greater use of SCM practices, which in turn results in increased co-benefits. SCM had a potentially balancing effect in the production potential and soil health as described below (loop 8, Table 2 and Fig. 4). Farms with lower production potential and less fertile soils may have less interest in adoption of SCM practices considering the land capability, however, SCM practices induced co-benefits and enhanced production potential along with improvements in soil health could lead to greater adoption of SCM practices.

## Discussion

The novel approach we took helped to identify the features that farmers and service providers consider influence SCM and the relationships, interactions and feedback loops between these features. The discussion is focused on the critical SES features and causality (interactions and feedback loops) of the current SES of SCM in the grazing regimes found in this study. The implications of the SES for SCM will be reflected in the context of local (farm production), national carbon policy (ERF), and international (SDGs) goals related to soil carbon sequestration.

Despite the existence of government carbon farming policies and incentives, highly experienced farmers are hesitant to make use of these incentives due to their opaqueness, with the result that the policies have had limited influence as a feature of the current SES of SCM. On the other hand, for farmers’ current SCM the features of farm production potential, SCM practices, training and education support, farmers’ social networks (e.g., Facebook groups, Landcare groups and other farmers), scientific support (e.g., soil testing), non-government organisation support (e.g., organising seminars and field days on SCM) and expert SCM information (Figs. 3 and 4) were important. Farmers were motivated to manage soil carbon irrespective of resource unit features (e.g., farm type, proprietorship, geographical location, farm size) because of the likely co-benefits of SCM under interaction-output first tier concept, such as improved soil health and farm production (grass and livestock). To improve the SCM network in this region there is a need to develop stronger information flows, improved connectivity to features and connections to government policies.

This study identified five broad findings that are relevant to soil carbon policy for a more inclusive agenda with improved information flows and greater incentives for other landholders to undertake SCM. These findings were: multiple co-benefits of SCM, inclusion of pluralistic values, valuing and funding training and education schemes, supporting farmers’ social networks, and understanding the importance of the SES feedback loops and their interactions for SCM. These broad findings highlights some of the strengths, weaknesses, opportunities and threats of the current SES of SCM in the grazing regimes of Australia, which are not necessarily mutually exclusive.

The SES highlights the critical role of co-benefits in SCM as an opportunity to reframe the narrative of soil carbon policy around this feature of farmers’ SCM. This was emphasised in two feedback loops from both the farmers’ and service providers’ perspective: (i) SCM enhances SCM co-benefits and leads to greater adoption of SCM (reinforcing feedback loop 7, Table 2); (ii) the additional cost of SCM (e.g., water infrastructure, fencing for grazing management) was compensated by the SCM co-benefits—improved soil health and production potential—which led to increased adoption of SCM across a farm (Dumbrell et al. 2016) (balancing feedback loop 3, Table 2). These feedback loops demonstrate the dynamism of the SES relationships and provide guidance for policymakers when considering the level and types of public and private investment. Moreover, it could provide opportunities for government to design its communication and incentive strategies for mitigating climate change to align better with farmers’ aspirations for SCM (Cohen et al. 2021).

The SES relationships and feedback loops (Table 2, Fig. 4) also identify the potential weaknesses of the existing soil carbon projects under ERF. The projects supported by ERF are governed by a centralised authority, using protocols designed to measure and monitor improvements in soil carbon (Australian Government 2020). The results of this study support the need for pluralism in climate change policymaking in terms of the range of stakeholders whose views contribute to policy, the suite of collateral benefits that may be needed to persuade targeted stakeholders beyond the direct objective of GHG sequestration (Cohen et al. 2021) and the array of motivations that drive farmer behaviours beyond economic advancement. Such co-production of policy is an opportunity that may positively influence farmers’ motivation to manage soil carbon at the farm level. Currently, a soil carbon project is designed to focus on a single feature (i.e., improving soil carbon), but does not consider the wider trade-offs and benefits for the whole soil carbon cycle. The farmers’ SES of SCM shows that income and lack of debt were not motivating factors for SCM, under current policy settings, and were weakly connected to many important features in the SES (Fig. 4). The dominant neoliberal paradigm has distanced government policy from farmers on the assumption that market forces will be sufficient to lead to change and adoption of SCM. Such an approach is a missed opportunity that may neither take into account nor harness the power of a range of farmer motivations to manage soil carbon.

A valued feature of farmers’ SES of SCM was training and education support, which was positively connected to SCM practices, production potential, co-benefits, soil health and trust (Table 2). Training and education support are not part of the existing soil carbon project scheme under ERF (Australian Government 2020). Nevertheless, the value of increased training and education support coupled with grants for on-farm SCM research in the SES for SCM would be to build trust in SCM information and soil carbon policies (Lobry de Bruyn et al. 2017). In addition through farmers knowledge sharing with other farmers there could be an improvement in information flows, with sources they find credible and trustworthy (Rust et al. 2021). As the farmers interviewed in this study were long-term practitioners of SCM the SES was well established, and could provide a pathway of SCM for other practitioners, especially those with less experience. The latest funding for soil extension and soil testing under National Soils Strategy in Australia (Australian Government 2021), with training as Registered Soil Practitioner recognises the need for reinvigoration of training support and upskilling of farmers for measuring soil carbon. Another valuable human resource is that of long-term practitioners of SCM and how to incorporate their experiences in future training programmes.

The strength of the current SES for SCM was farmers’ social networks, but they are also under threat as there is a lack of support for, and recognition of farmers’ informal and formal social networks by government. The SES relationships anticipate the potential benefits of closer interaction amongst farmers’ social networks, training and education and the governance features of soil carbon policy. Training, education and information on practice-oriented carbon pricing (e.g., schemes for long-term practitioners who have established SCM) and monitoring mechanisms flowing through social networks (Kragt et al. 2017) could build farmers’ trust in soil carbon policy and ensure greater engagement with it. Many of the existing social networks, largely supported by Landcare, are less active than they once were as a social network and require reinvigoration. As social networks are an important feature of SCM, government needs to reinvigorate the social networks (Jones et al. 2019), especially those that are aging or inactive (Lobry de Bruyn and Andrews 2016), by providing incentives or pathways for connecting to those extension agents or independent advisors who already have strong relationships with practitioners as part of their current level of practice.

The identified feedback loops and SES relationships (Table 2 and Fig. 4) showed the strength of the SCM in the grazing regimes by considering the whole SES of SCM. SCM reinforces the positive change of income within a manageable planning time frame (Amin et al. 2023) (loop 4, Table 2), and communicating those achievements through farmers’ social networks reinforces the positive outcomes of SCM (loops 5 and 9, Table 2). However, the change of income due to increased farm production created a trade-off between farm production and GHG emissions (methane and carbon dioxide). Only if cattle numbers remained static and do not increase in the managed grazing lands would result in the sink switching to a source (Chang et al. 2021). Essentially, SCM cost and farm debt were compensated by the outcomes of SCM such as co-benefits (loops 1 and 10, Table 2) but at the cost of additional agricultural GHG emissions. Such increases in GHG emissions from SCM call for improved management practices to retain carbon in soil (Whitehead 2020), consideration of rotational grazing (Liu et al. 2020) and reduced stocking rates (Bork et al. 2020; Chang et al. 2021) to achieve the government net zero emissions target from this sector. Overall, for a successful soil carbon policy, the interrelationships between the feedback loops show the importance of focusing on the whole SES for SCM rather than particular relationships.

The consolidated SES for SCM (Fig. 4) suggests that soil carbon projects under current government policy need to be more deeply connected with other influential SES features of SCM including: co-benefits, social networks and training and education support. The policy design also needs to consider a whole system approach and inclusive participation (i.e., current ERF schemes excludes long-term practitioners) to achieve improvements in soil carbon offsets.

The research undertook cross-sectional analysis in a qualitative manner due to unavailability of time series data and confidentiality of the existing farm-level data (e.g., socio-economic, soil test data). The SES relationships were constructed from the lived experience of farmers who have managed their land for decades and may well reflect the SES features of other grazing systems elsewhere in Australia and possibly internationally with similar demographics and environments. The participant selection process of this study was purposive (e.g., including long-term SCM practitioners of rotational grazing), thus farmers not currently practising SCM (e.g., through rotational grazing) and their SES were not considered in this study. The relevance of the SES of SCM for their SCM behaviour would also need to be investigated to understand if the same features would motivate their actions in SCM, and if the feedback loops would operate in a similar manner. SES of this study is based on a small subset of farmers they represent highly skilled, self-taught and long-term practitioners of rotational grazing. Although their SES of current SCM may not reflect the wider community of graziers it does provides a perspective on what contributes to SCM and what does not help them in their current system. For the service providers, it represents what they think is the SES for graziers more generally, but again those SES features originate from the interviewed farmers. The SES features and interrelationships could guide others to explore the challenges and current needs of stakeholders who are engaged in rotational grazing or are seeking to be to achieve important local, national and international objectives, such as SCM co-benefits, GHG reduction, carbon sequestration targets and SDGs, respectively. In general, the methodology of the study could be useful for operationalising the SES framework to other SCM SESs.

## Conclusion

We used a novel approach of operationalising Ostrom’s SES framework by combining qualitative and quantitative methods to identify the SES relationships in SCM in a high-rainfall grazing region of Australia. Our SES for SCM shows the relative importance of each SCM feature in the system from the farmers’ perspectives, and revealed that farmers place high importance on the features of training and education support, social networks, and co-benefits, which are currently inadequately addressed in existing Australian policy mechanisms such as the ERF. This study revealed the weak connectivity of the current SCM features in the studied system and indicated multiple foci for building a more highly connected SCM network that could be carbon neutral by 2050. The potential feedback loops identified in this study could provide guidance to policymakers to improve the SCM system in Australia so that it can meet not only the farmers’ requirements to achieve the identified co-benefits of SCM but also the government’s goal of improved soil carbon sequestration that can offset GHG emissions. Our approach to studying the SES for SCM would be useful in similar data-poor regions of the world.

## Supplementary Information

Supplementary Information
