# Supplementary material for: The Social-Ecological System of Farmers’ Current Soil Carbon Management in Australian Grazing Lands
Source: Environ Manage. 2023 Mar 7;72(2):294–308. doi: 10.1007/s00267-023-01801-4 (PMC10287766; doi:10.1007/s00267-023-01801-4)
Supplement: Supplementary file 1 — Supplementary Information [file 267_2023_1801_MOESM1_ESM.docx]

**Supplementary Information**

**SI Table 1.** Network/relationship of the SCM features with SCM outcome at farm level (SCM1) in the grazing lands of Northern Tablelands***

| 1^st^ tier concept | SES features | SCM Outcome | Response  (With influence= 1, no influence = 0) |
| --- | --- | --- | --- |
| Resource system | Geographical location | pH level | 0 |
|  | Geographical location | Soil moisture | 1 |
|  | Geographical location | Soil structure | 1 |
|  | Geographical location | Soil biodiversity | 0 |
|  | Geographical location | Landscape aesthetics | 1 |
|  | Geographical location | Soil water holding capacity | 1 |
|  | Geographical location | Soil erosion | 1 |
|  | Geographical location | Soil nutrients | 1 |
|  | Geographical location | Soil carbon content | 1 |
|  | Geographical location | Mental health | 1 |
|  | Geographical location | Shelter for livestock | 1 |
|  | Small Farm | pH level | 0 |
|  | Small Farm | Soil moisture | 1 |
|  | Small Farm | Soil structure | 1 |
|  | Small Farm | Soil biodiversity | 0 |
|  | Small Farm | Landscape aesthetics | 1 |
|  | Small Farm | Soil water holding capacity | 1 |
|  | Small Farm | Soil erosion | 1 |
|  | Small Farm | Soil nutrients | 1 |
|  | Small Farm | Soil carbon content | 1 |
|  | Small Farm | Mental health | 1 |
|  | Small Farm | Shelter for livestock | 1 |
|  | Grazing | pH level | 0 |
|  | Grazing | Soil moisture | 1 |
|  | Grazing | Soil structure | 1 |
|  | Grazing | Soil biodiversity | 0 |
|  | Grazing | Landscape aesthetics | 1 |
|  | Grazing | Soil water holding capacity | 1 |
|  | Grazing | Soil erosion | 1 |
|  | Grazing | Soil nutrients | 1 |
|  | Grazing | Soil carbon content | 1 |
|  | Grazing | Mental health | 1 |
|  | Grazing | Shelter for livestock | 1 |
|  | Family farm | pH level | 0 |
|  | Family farm | Soil moisture | 1 |
|  | Family farm | Soil structure | 1 |
|  | Family farm | Soil biodiversity | 0 |
|  | Family farm | Landscape aesthetics | 1 |
|  | Family farm | Soil water holding capacity | 1 |
|  | Family farm | Soil erosion | 1 |
|  | Family farm | Soil nutrients | 1 |
|  | Family farm | Soil carbon content | 1 |
|  | Family farm | Mental health | 1 |
|  | Family farm | Shelter for livestock | 1 |
|  | No loan | pH level | 0 |
|  | No loan | Soil moisture | 1 |
|  | No loan | Soil structure | 1 |
|  | No loan | Soil biodiversity | 0 |
|  | No loan | Landscape aesthetics | 1 |
|  | No loan | Soil water holding capacity | 1 |
|  | No loan | Soil erosion | 1 |
|  | No loan | Soil nutrients | 1 |
|  | No loan | Soil carbon content | 1 |
|  | No loan | Mental health | 1 |
|  | No loan | Shelter for livestock | 1 |
| Resource unit | Production potential | pH level | 0 |
|  | Production potential | Soil moisture | 1 |
|  | Production potential | Soil structure | 1 |
|  | Production potential | Soil biodiversity | 0 |
|  | Production potential | Landscape aesthetics | 1 |
|  | Production potential | Soil water holding capacity | 1 |
|  | Production potential | Soil erosion | 1 |
|  | Production potential | Soil nutrients | 1 |
|  | Production potential | Soil carbon content | 1 |
|  | Production potential | Mental health | 1 |
|  | Production potential | Shelter for livestock | 1 |
|  | Soil health | pH level | 0 |
|  | Soil health | Soil moisture | 1 |
|  | Soil health | Soil structure | 1 |
|  | Soil health | Soil biodiversity | 0 |
|  | Soil health | Landscape aesthetics | 1 |
|  | Soil health | Soil water holding capacity | 1 |
|  | Soil health | Soil erosion | 1 |
|  | Soil health | Soil nutrients | 1 |
|  | Soil health | Soil carbon content | 1 |
|  | Soil health | Mental health | 1 |
|  | Soil health | Shelter for livestock | 1 |
|  | SCM practices | pH level | 0 |
|  | SCM practices | Soil moisture | 1 |
|  | SCM practices | Soil structure | 1 |
|  | SCM practices | Soil biodiversity | 0 |
|  | SCM practices | Landscape aesthetics | 1 |
|  | SCM practices | Soil water holding capacity | 1 |
|  | SCM practices | Soil erosion | 1 |
|  | SCM practices | Soil nutrients | 1 |
|  | SCM practices | Soil carbon content | 1 |
|  | SCM practices | Mental health | 1 |
|  | SCM practices | Shelter for livestock | 1 |
|  | Climate | pH level | 0 |
|  | Climate | Soil moisture | 1 |
|  | Climate | Soil structure | 1 |
|  | Climate | Soil biodiversity | 0 |
|  | Climate | Landscape aesthetics | 1 |
|  | Climate | Soil water holding capacity | 1 |
|  | Climate | Soil erosion | 1 |
|  | Climate | Soil nutrients | 1 |
|  | Climate | Soil carbon content | 1 |
|  | Climate | Mental health | 1 |
|  | Climate | Shelter for livestock | 1 |
|  | Change of income | pH level | 0 |
|  | Change of income | Soil moisture | 1 |
|  | Change of income | Soil structure | 1 |
|  | Change of income | Soil biodiversity | 0 |
|  | Change of income | Landscape aesthetics | 1 |
|  | Change of income | Soil water holding capacity | 1 |
|  | Change of income | Soil erosion | 1 |
|  | Change of income | Soil nutrients | 1 |
|  | Change of income | Soil carbon content | 1 |
|  | Change of income | Mental health | 1 |
|  | Change of income | Shelter for livestock | 1 |
|  | Agri-environmental benefits | pH level | 0 |
|  | Agri-environmental benefits | Soil moisture | 1 |
|  | Agri-environmental benefits | Soil structure | 1 |
|  | Agri-environmental benefits | Soil biodiversity | 0 |
|  | Agri-environmental benefits | Landscape aesthetics | 1 |
|  | Agri-environmental benefits | Soil water holding capacity | 1 |
|  | Agri-environmental benefits | Soil erosion | 1 |
|  | Agri-environmental benefits | Soil nutrients | 1 |
|  | Agri-environmental benefits | Soil carbon content | 1 |
|  | Agri-environmental benefits | Mental health | 1 |
|  | Agri-environmental benefits | Shelter for livestock | 1 |
|  | SCM cost | pH level | 0 |
|  | SCM cost | Soil moisture | 1 |
|  | SCM cost | Soil structure | 1 |
|  | SCM cost | Soil biodiversity | 0 |
|  | SCM cost | Landscape aesthetics | 1 |
|  | SCM cost | Soil water holding capacity | 1 |
|  | SCM cost | Soil erosion | 1 |
|  | SCM cost | Soil nutrients | 1 |
|  | SCM cost | Soil carbon content | 1 |
|  | SCM cost | Mental health | 1 |
|  | SCM cost | Shelter for livestock | 1 |
| Governance | Govt org | pH level | 0 |
|  | Govt org | Soil moisture | 0 |
|  | Govt org | Soil structure | 0 |
|  | Govt org | Soil biodiversity | 0 |
|  | Govt org | Landscape aesthetics | 0 |
|  | Govt org | Soil water holding capacity | 0 |
|  | Govt org | Soil erosion | 0 |
|  | Govt org | Soil nutrients | 0 |
|  | Govt org | Soil carbon content | 0 |
|  | Govt org | Mental health | 0 |
|  | Govt org | Shelter for livestock | 0 |
|  | Non Govt org | pH level | 0 |
|  | Non Govt org | Soil moisture | 1 |
|  | Non Govt org | Soil structure | 1 |
|  | Non Govt org | Soil biodiversity | 0 |
|  | Non Govt org | Landscape aesthetics | 1 |
|  | Non Govt org | Soil water holding capacity | 1 |
|  | Non Govt org | Soil erosion | 1 |
|  | Non Govt org | Soil nutrients | 1 |
|  | Non Govt org | Soil nutrient cycling | 1 |
|  | Non Govt org | Soil carbon content | 1 |
|  | Non Govt org | Mental health | 1 |
|  | Non Govt org | Shelter for livestock | 1 |
|  | Farm research grant | pH level | 0 |
|  | Farm research grant | Soil moisture | 1 |
|  | Farm research grant | Soil structure | 1 |
|  | Farm research grant | Soil biodiversity | 0 |
|  | Farm research grant | Landscape aesthetics | 1 |
|  | Farm research grant | Soil water holding capacity | 1 |
|  | Farm research grant | Soil erosion | 1 |
|  | Farm research grant | Soil nutrients | 1 |
|  | Farm research grant | Soil carbon content | 1 |
|  | Farm research grant | Mental health | 1 |
|  | Farm research grant | Shelter for livestock | 1 |
|  | Scientific support | pH level | 0 |
|  | Scientific support | Soil moisture | 1 |
|  | Scientific support | Soil structure | 1 |
|  | Scientific support | Soil biodiversity | 0 |
|  | Scientific support | Landscape aesthetics | 1 |
|  | Scientific support | Soil water holding capacity | 1 |
|  | Scientific support | Soil erosion | 1 |
|  | Scientific support | Soil nutrients | 1 |
|  | Scientific support | Soil carbon content | 1 |
|  | Scientific support | Mental health | 1 |
|  | Scientific support | Shelter for livestock | 1 |
|  | Government investments | pH level | 0 |
|  | Government investments | Soil moisture | 1 |
|  | Government investments | Soil structure | 1 |
|  | Government investments | Soil biodiversity | 0 |
|  | Government investments | Landscape aesthetics | 1 |
|  | Government investments | Soil water holding capacity | 1 |
|  | Government investments | Soil erosion | 1 |
|  | Government investments | Soil nutrients | 1 |
|  | Government investments | Soil carbon content | 1 |
|  | Government investments | Mental health | 1 |
|  | Government investments | Shelter for livestock | 1 |
|  | Private investments | pH level | 0 |
|  | Private investments | Soil moisture | 1 |
|  | Private investments | Soil structure | 1 |
|  | Private investments | Soil biodiversity | 0 |
|  | Private investments | Landscape aesthetics | 1 |
|  | Private investments | Soil water holding capacity | 1 |
|  | Private investments | Soil erosion | 1 |
|  | Private investments | Soil nutrients | 1 |
|  | Private investments | Soil nutrient cycling | 1 |
|  | Private investments | Soil carbon content | 1 |
|  | Private investments | Mental health | 1 |
|  | Private investments | Shelter for livestock | 0 |
|  | Carbon pricing and monitoring | pH level | 0 |
|  | Carbon pricing and monitoring | Soil moisture | 1 |
|  | Carbon pricing and monitoring | Soil structure | 1 |
|  | Carbon pricing and monitoring | Soil biodiversity | 0 |
|  | Carbon pricing and monitoring | Landscape aesthetics | 1 |
|  | Carbon pricing and monitoring | Soil water holding capacity | 1 |
|  | Carbon pricing and monitoring | Soil erosion | 1 |
|  | Carbon pricing and monitoring | Soil nutrients | 1 |
|  | Carbon pricing and monitoring | Soil carbon content | 1 |
|  | Carbon pricing and monitoring | Mental health | 1 |
|  | Carbon pricing and monitoring | Shelter for livestock | 1 |
|  | Certainty of payment | pH level | 0 |
|  | Certainty of payment | Soil moisture | 0 |
|  | Certainty of payment | Soil structure | 0 |
|  | Certainty of payment | Soil biodiversity | 0 |
|  | Certainty of payment | Landscape aesthetics | 0 |
|  | Certainty of payment | Soil water holding capacity | 0 |
|  | Certainty of payment | Soil erosion | 0 |
|  | Certainty of payment | Soil nutrients | 0 |
|  | Certainty of payment | Soil carbon content | 0 |
|  | Certainty of payment | Mental health | 0 |
|  | Certainty of payment | Shelter for livestock | 0 |
|  | Training and education supports | pH level | 0 |
|  | Training and education supports | Soil moisture | 1 |
|  | Training and education supports | Soil structure | 1 |
|  | Training and education supports | Soil biodiversity | 0 |
|  | Training and education supports | Landscape aesthetics | 1 |
|  | Training and education supports | Soil water holding capacity | 1 |
|  | Training and education supports | Soil erosion | 1 |
|  | Training and education supports | Soil nutrients | 1 |
|  | Training and education supports | Soil carbon content | 1 |
|  | Training and education supports | Mental health | 1 |
|  | Training and education supports | Shelter for livestock | 1 |
|  | Expert information | pH level | 0 |
|  | Expert information | Soil moisture | 1 |
|  | Expert information | Soil structure | 1 |
|  | Expert information | Soil biodiversity | 0 |
|  | Expert information | Landscape aesthetics | 1 |
|  | Expert information | Soil water holding capacity | 1 |
|  | Expert information | Soil erosion | 1 |
|  | Expert information | Soil nutrients | 1 |
|  | Expert information | Soil carbon content | 1 |
|  | Expert information | Mental health | 1 |
|  | Expert information | Shelter for livestock | 1 |
|  | Soil carbon policy | pH level | 0 |
|  | Soil carbon policy | Soil moisture | 0 |
|  | Soil carbon policy | Soil structure | 0 |
|  | Soil carbon policy | Soil biodiversity | 0 |
|  | Soil carbon policy | Landscape aesthetics | 0 |
|  | Soil carbon policy | Soil water holding capacity | 0 |
|  | Soil carbon policy | Soil erosion | 0 |
|  | Soil carbon policy | Soil nutrients | 0 |
|  | Soil carbon policy | Soil carbon content | 0 |
|  | Soil carbon policy | Mental health | 0 |
|  | Soil carbon policy | Shelter for livestock | 0 |
|  | Social network | pH level | 0 |
|  | Social network | Soil moisture | 0 |
|  | Social network | Soil structure | 0 |
|  | Social network | Soil biodiversity | 0 |
|  | Social network | Landscape aesthetics | 0 |
|  | Social network | Soil water holding capacity | 0 |
|  | Social network | Soil erosion | 0 |
|  | Social network | Soil nutrients | 0 |
|  | Social network | Soil carbon content | 0 |
|  | Social network | Mental health | 0 |
|  | Social network | Shelter for livestock | 0 |
|  | Trusted experts network | pH level | 0 |
|  | Trusted experts network | Soil moisture | 1 |
|  | Trusted experts network | Soil structure | 1 |
|  | Trusted experts network | Soil biodiversity | 0 |
|  | Trusted experts network | Landscape aesthetics | 1 |
|  | Trusted experts network | Soil water holding capacity | 1 |
|  | Trusted experts network | Soil erosion | 1 |
|  | Trusted experts network | Soil nutrients | 1 |
|  | Trusted experts network | Soil carbon content | 1 |
|  | Trusted experts network | Mental health | 1 |
|  | Trusted experts network | Shelter for livestock | 1 |
| Actors | Govt officer | pH level | 0 |
|  | Govt officer | Soil moisture | 0 |
|  | Govt officer | Soil structure | 0 |
|  | Govt officer | Soil biodiversity | 0 |
|  | Govt officer | Landscape aesthetics | 0 |
|  | Govt officer | Soil water holding capacity | 0 |
|  | Govt officer | Soil erosion | 0 |
|  | Govt officer | Soil nutrients | 0 |
|  | Govt officer | Soil nutrient cycling | 0 |
|  | Govt officer | Soil carbon content | 0 |
|  | Govt officer | Mental health | 0 |
|  | Govt officer | Shelter for livestock | 0 |
|  | Independent advisors | pH level | 0 |
|  | Independent advisors | Soil moisture | 1 |
|  | Independent advisors | Soil structure | 1 |
|  | Independent advisors | Soil biodiversity | 1 |
|  | Independent advisors | Landscape aesthetics | 1 |
|  | Independent advisors | Soil water holding capacity | 1 |
|  | Independent advisors | Soil erosion | 1 |
|  | Independent advisors | Soil nutrients | 1 |
|  | Independent advisors | Soil nutrient cycling | 1 |
|  | Independent advisors | Soil carbon content | 1 |
|  | Independent advisors | Mental health | 1 |
|  | Independent advisors | Shelter for livestock | 1 |
|  | Farmers | pH level | 0 |
|  | Farmers | Soil moisture | 1 |
|  | Farmers | Soil structure | 1 |
|  | Farmers | Soil biodiversity | 0 |
|  | Farmers | Landscape aesthetics | 1 |
|  | Farmers | Soil water holding capacity | 1 |
|  | Farmers | Soil erosion | 1 |
|  | Farmers | Soil nutrients | 1 |
|  | Farmers | Soil nutrient cycling | 1 |
|  | Farmers | Soil carbon content | 1 |
|  | Farmers | Mental health | 1 |
|  | Farmers | Shelter for livestock | 1 |
|  | Scientists | pH level | 0 |
|  | Scientists | Soil moisture | 1 |
|  | Scientists | Soil structure | 1 |
|  | Scientists | Soil biodiversity | 0 |
|  | Scientists | Landscape aesthetics | 1 |
|  | Scientists | Soil water holding capacity | 1 |
|  | Scientists | Soil erosion | 1 |
|  | Scientists | Soil nutrients | 1 |
|  | Scientists | Soil nutrient cycling | 1 |
|  | Scientists | Soil carbon content | 1 |
|  | Scientists | Mental health | 1 |
|  | Scientists | Shelter for livestock | 1 |
|  | Education institute | pH level | 0 |
|  | Education institute | Soil moisture | 1 |
|  | Education institute | Soil structure | 1 |
|  | Education institute | Soil biodiversity | 0 |
|  | Education institute | Landscape aesthetics | 1 |
|  | Education institute | Soil water holding capacity | 1 |
|  | Education institute | Soil erosion | 1 |
|  | Education institute | Soil nutrients | 1 |
|  | Education institute | Soil nutrient cycling | 1 |
|  | Education institute | Soil carbon content | 1 |
|  | Education institute | Mental health | 1 |
|  | Education institute | Shelter for livestock | 1 |
|  | SCM attitude | pH level | 0 |
|  | SCM attitude | Soil moisture | 1 |
|  | SCM attitude | Soil structure | 1 |
|  | SCM attitude | Soil biodiversity | 0 |
|  | SCM attitude | Landscape aesthetics | 1 |
|  | SCM attitude | Soil water holding capacity | 1 |
|  | SCM attitude | Soil erosion | 1 |
|  | SCM attitude | Soil nutrients | 1 |
|  | SCM attitude | Soil nutrient cycling | 1 |
|  | SCM attitude | Soil carbon content | 1 |
|  | SCM attitude | Mental health | 1 |
|  | SCM attitude | Shelter for livestock | 1 |
|  | Soil stewardship ethics | pH level | 0 |
|  | Soil stewardship ethics | Soil moisture | 1 |
|  | Soil stewardship ethics | Soil structure | 1 |
|  | Soil stewardship ethics | Soil biodiversity | 0 |
|  | Soil stewardship ethics | Landscape aesthetics | 1 |
|  | Soil stewardship ethics | Soil water holding capacity | 1 |
|  | Soil stewardship ethics | Soil erosion | 1 |
|  | Soil stewardship ethics | Soil nutrients | 1 |
|  | Soil stewardship ethics | Soil nutrient cycling | 1 |
|  | Soil stewardship ethics | Soil carbon content | 1 |
|  | Soil stewardship ethics | Mental health | 1 |
|  | Soil stewardship ethics | Shelter for livestock | 1 |
|  | Technologies available | pH level | 0 |
|  | Technologies available | Soil moisture | 0 |
|  | Technologies available | Soil structure | 0 |
|  | Technologies available | Soil biodiversity | 0 |
|  | Technologies available | Landscape aesthetics | 0 |
|  | Technologies available | Soil water holding capacity | 0 |
|  | Technologies available | Soil erosion | 0 |
|  | Technologies available | Soil nutrients | 0 |
|  | Technologies available | Soil nutrient cycling | 0 |
|  | Technologies available | Soil carbon content | 0 |
|  | Technologies available | Mental health | 0 |
|  | Technologies available | Shelter for livestock | 0 |
|  | Trust | pH level | 0 |
|  | Trust | Soil moisture | 0 |
|  | Trust | Soil structure | 0 |
|  | Trust | Soil biodiversity | 0 |
|  | Trust | Landscape aesthetics | 0 |
|  | Trust | Soil water holding capacity | 0 |
|  | Trust | Soil erosion | 0 |
|  | Trust | Soil nutrients | 0 |
|  | Trust | Soil nutrient cycling | 0 |
|  | Trust | Soil carbon content | 0 |
|  | Trust | Mental health | 0 |
|  | Trust | Shelter for livestock | 0 |

***Notes: The assigned value here is for farm 1 (SCM1). The study has produced a table for each farmer interview (n=25) for the network analysis.

**SI Table 2.** Farming experience, SCM practices of the interviewed farmers and farm soil types.

| Farm No. | Farmer Age | Total farm experience | Farm experience in SCM practice at current location | Farm area (ha) | Soil type parent material | Soil carbon management practices | | | | | | | | |
| --- | --- | --- | --- | --- | --- | --- | --- | --- | --- | --- | --- | --- | --- | --- |
|  |  |  |  |  |  | Mulching | Native vegetation establishment | Increasing pasture area | Intercropping with perennial pasture | Stubble retention | Legume in pasture | Tree Planting | Bio-char | Grazing system |
| SCM1 | 65 | 40 | 29 | 40 | Sedimentary | No | No | No | No | No | No | Yes | No | Strategic |
| SCM2 | 66 | 45 | 40 | 3350 | Granite | Yes | Yes | Yes | Yes | Yes | Yes | No | No | Rotational |
| SCM3 | 56 | 8 | 8 | 1250 | Granite | Yes | No | No | No | No | Yes | Yes | No | Rotational |
| SCM4 | 69 | 50 | 40 | 3000 | Basalt | No | Yes | No | No | No | No | No | No | Rotational |
| SCM5 | 59 | 40 | 36 | 120 | Basalt | Yes | No | Yes | No | Yes | Yes | Yes | No | Rotational |
| SCM6 | 40 | 25 | 8 | 400 | Sedimentary | No | No | No | No | No | No | Yes | No | Rotational |
| SCM7 | 59 | 40 | 40 | 1450 | Sedimentary | No | No | No | No | No | Yes | Yes | No | Rotational |
| SCM8 | 61 | 40 | 40 | 999 | Sedimentary | No | No | No | No | No | No | Yes | No | Rotational |
| SCM9 | 68 | 48 | 48 | 1202 | Granite | No | No | No | No | No | Yes | Yes | No | Strategic |
| SCM10 | 61 | 12 | 10 | 923 | Basalt | No | No | No | No | No | Yes | No | No | Rotational |
| SCM11 | 73 | 60 | 30 | 2000 | Basalt | Yes | No | No | Yes | No | Yes | Yes | No | Rotational |
| SCM12 | 55 | 36 | 12 | 1400 | Basalt | No | No | No | No | Yes | Yes | Yes | No | Rotational |
| SCM13 | 51 | 30 | 15 | 8 | Granite | No | No | No | No | No | No | Yes | No | Rotational |
| SCM14 | 45 | 25 | 25 | 1500 | Granite | No | No | No | No | No | Yes | Yes | No | Rotational |
| SCM15 | 64 | 40 | 20 | 260 | Basalt | Yes | No | Yes | No | No | Yes | Yes | No | Rotational |
| SCM16 | 64 | 40 | 10 | 1620 | Basalt | No | No | No | No | No | No | Yes | No | Rotational |
| SCM17 | 71 | 51 | 5 | 220 | Basalt | Yes | No | No | No | No | Yes | Yes | Yes | Rotational |
| SCM18 | 71 | 50 | 16 | 45 | Basalt | Yes | No | No | No | Yes | Yes | No | No | Time control |
| SCM19 | 54 | 25 | 5 | 64 | Granite | No | No | No | Yes | No | No | Yes | No | Rotational |
| SCM20 | 58 | 10 | 9 | 454 | Basalt | Yes | No | No | No | No | No | No | No | Rotational |
| SCM21 | 42 | 17 | 6 | 527 | Basalt | No | Yes | No | No | No | No | No | No | Rotational |
| SCM22 | 82 | 64 | 64 | 2134 | Granite | Yes | No | No | No | No | Yes | No | No | Rotational |
| SCM23 | 55 | 5 | 3 | 43 | Granite | Yes | No | No | No | Yes | No | Yes | No | Rotational |
| SCM24 | 62 | 15 | 15 | 2626 | Granite | No | No | Yes | Yes | No | No | Yes | No | Rotational |
| SCM25 | 69 | 55 | 55 | 530 | Granite | No | No | No | No | Yes | Yes | No | No | Rotational |
| Total= 25 farms | Average= 60 years | Average =35 years | Average= 24 years | Average= 1047 ha |  |  |  |  |  |  |  |  |  |  |

**SI Table 3.** Interview responses on the features of SCM at farm level (n = 25 farms)

| First tier concepts | Second-tier or features | With influence= 1 (%) | No influence= 0 (%) |
| --- | --- | --- | --- |
| Resource system | Geographical location | 72 | 28 |
|  | Large farm | 52 | 48 |
|  | Small farm | 48 | 52 |
|  | Grazing farm | 76 | 24 |
|  | Mixed farm | 24 | 76 |
|  | Company Farm | 12 | 88 |
|  | Family farm | 88 | 12 |
|  | No loan farm | 40 | 60 |
|  | Loan Farm | 60 | 40 |
|  | Fertile soil | 40 | 60 |
|  | Less fertile soil | 60 | 40 |
|  | Soil health | 88 | 12 |
| Resource unit | Production potential | 100 | 0 |
|  | SCM practices | 92 | 8 |
|  | Climate | 100 | 0 |
|  | Change of income | 84 | 16 |
|  | Agri-environmental benefits | 92 | 8 |
|  | SCM cost | 100 | 0 |
| Governance | Govt org | 32 | 68 |
|  | Non Govt org | 88 | 12 |
|  | Farm research grant | 56 | 44 |
|  | Scientific support | 100 | 0 |
|  | Govt investments | 24 | 76 |
|  | Private investments | 40 | 60 |
|  | Carbon pricing and monitoring | 48 | 52 |
|  | Certainty of payment | 60 | 40 |
|  | Training and education supports | 96 | 4 |
|  | Expert information | 96 | 4 |
|  | Soil carbon policy | 56 | 44 |
|  | Social network | 88 | 12 |
|  | Trusted experts network | 84 | 16 |
| Actors | Govt officer | 12 | 88 |
|  | Independent advisors | 96 | 4 |
|  | Farmers | 100 | 0 |
|  | Scientists | 92 | 8 |
|  | Education institute | 60 | 40 |
|  | SCM attitude | 80 | 20 |
|  | Soil stewardship ethics | 68 | 32 |
|  | Technologies available | 32 | 68 |
|  | Trust | 88 | 12 |
| Interaction-Output | pH level | 40 | 60 |
|  | Soil moisture | 100 | 0 |
|  | Soil structure | 92 | 8 |
|  | Soil biodiversity | 80 | 20 |
|  | Landscape aesthetics | 64 | 36 |
|  | Soil water holding capacity | 76 | 24 |
|  | Soil erosion | 72 | 28 |
|  | Soil nutrients | 68 | 32 |
|  | Soil carbon content | 80 | 20 |
|  | Mental health | 40 | 60 |
|  | Shelter for livestock | 72 | 28 |

**SI Table 4.** Summary of positive interactions identified by Farmer workshops

| **These features** | ***positively* influenced these features** |
| --- | --- |
| - Co-benefits of SCM – Improved soil moisture, nutrients, water holding capacity and soil structure) | - Production potential - Soil health - Support of other farmers for SCM - Interest in training & educational |
| - Co-benefits of SCM – Mental health and landscape aesthetics | - Interest in SCM practices |
| - Agri-environmental benefits of SCM | - Production potential of the farm - Soil health |
| - Training & education | - Other farmers not yet involved in SCM |
| - Soil stewardship ethic | - SCM - Co-benefits from SCM |
| - Trust between actors and strong soil stewardship | - Interest in and adoption of SCM, which in turn could improve income, leading to further investment in SCM |
| - Farmers’ social network | - SCM - Interest in training and education - Mental health |

**SI Table 5.** Summary of positive interactions identified by Service Provider workshops

| **These features** | ***positively* influenced these features** |
| --- | --- |
| SCM practices | Co-benefits of SCM |
| - Soil stewardship ethic | - Participation of other farmers who were not undertaking SCM and seeking grants for on-farm research - Support from government & non-government organizations - Seeking reliable scientific information - Participation in social networks |
| - Certainty of payment | - Cost of SCM - Income |
| - Effective carbon pricing and monitoring | - Change of income, which might result a positive effect on the debt status and lead to more adoption of SCM |
| - Government Investment - Private investment - Grants for on-fam research | - Availability and adoption of technology, which ultimately influences SCM |
| - Government officers - Training & education - Education institutes - Scientists | - Trust - Soil stewardship ethic - Farmers’ social network - Mainstream media |

**SI Table 6.** Summary of mixed interactions identified by Farmer (F) and Service Provider (SP) workshops

| **Group** | **These features** | **either *positively* or *negatively* influenced these features** |
| --- | --- | --- |
| F | - SCM practices | - Change of income, depending on how quickly a change resulted in benefits |
| F | - Non-government organizations’ support - Scientific support - Independent advisors - Grants for on-farm research, | - SCM, depending on whether the interaction between actors either built or eroded farmers’ trust and confidence in SCM |
| SP | - Carbon pricing and monitoring | - Soil carbon policy, depending on the extent to which policy was practice-oriented and based on land use and management. |

**SI Table 7.** Summary of negative interactions identified by Farmer workshops

| **These features** | ***negatively* influenced these features** |
| --- | --- |
| - Current soil carbon policy - Carbon pricing & monitoring - Lack of available technology | - SCM |
| - Farmers’ social network | - SCM |

**SI Table 8.** List of thematic nodes from interview

| Sl No. | Thematic nodes from interview |
| --- | --- |
| 1 | Actors |
| 2 | Main function of the group or organization |
| 3 | People or group involved |
| 4 | Reason for nominating the group |
| 5 | Social and cultural factors of influence |
| 6 | Social barrier for land management |
| 7 | Additional cost of carbon farm management |
| 8 | Benefits of carbon management |
| 9 | Best SCM |
| 10 | Carbon content |
| 11 | Existing challenge of SCM |
| 12 | Expectations |
| 13 | Farming system |
| 14 | Governance |
| 15 | Carbon credit |
| 16 | Carbon management insurance coverage |
| 17 | Eligibility of the government financial incentives |
| 18 | Farmer social network |
| 19 | Govt. carbon policy informed |
| 20 | Influence of govt. org. or individual |
| 21 | Main support to land management |
| 22 | Support of the government org or individual |
| 23 | Support of the private org or individual |
| 24 | Information system |
| 25 | Interaction and outcome |
| 26 | Land cover |
| 27 | Rainfall |
| 28 | Resource system |
| 29 | Resource Unit |
| 30 | Approach of management practice on farm |
| 31 | External scientific sources |
| 32 | Own research |
| 33 | Usual practice as before |
| 34 | Best Management for SOM |
| 35 | Effect on income |
| 36 | Environmental factors influence |
| 37 | Financial support for land management |
| 38 | Reason of management practice |
| 39 | SOM content in farm soil |
| 40 | Training for land management |
| 41 | System understanding |

**SI Table 9.** Two mode network/relationship of the SCM features at farm level (SCM 1- SCM25) in the grazing lands of Northern Tablelands

| **SCM features or second-tier** | **Farm Name** | | | | | | | | | | | | | | | | | | | | | | | | |
| --- | --- | --- | --- | --- | --- | --- | --- | --- | --- | --- | --- | --- | --- | --- | --- | --- | --- | --- | --- | --- | --- | --- | --- | --- | --- |
|  | **SCM 1** | **SCM 2** | **SCM 3** | **SCM 4** | **SCM 5** | **SCM 6** | **SCM 7** | **SCM 8** | **SCM 9** | **SCM 10** | **SCM 11** | **SCM 12** | **SCM 13** | **SCM 14** | **SCM 15** | **SCM 16** | **SCM 17** | **SCM 18** | **SCM 19** | **SCM 20** | **SCM 21** | **SCM 22** | **SCM 23** | **SCM 24** | **SCM 25** |
| Geographical location | 1 | 1 | 1 | 1 | 0 | 0 | 1 | 1 | 1 | 0 | 1 | 1 | 1 | 1 | 1 | 1 | 1 | 1 | 1 | 1 | 0 | 0 | 0 | 0 | 1 |
| Large farm | 0 | 1 | 1 | 1 | 0 | 0 | 0 | 1 | 1 | 1 | 1 | 1 | 0 | 1 | 1 | 1 | 0 | 0 | 0 | 0 | 0 | 1 | 0 | 1 | 0 |
| Small farm | 1 | 0 | 0 | 0 | 1 | 1 | 1 | 0 | 0 | 0 | 0 | 0 | 1 | 0 | 0 | 0 | 1 | 1 | 1 | 1 | 1 | 0 | 1 | 0 | 1 |
| Grazing farm | 1 | 0 | 1 | 1 | 0 | 0 | 0 | 1 | 1 | 1 | 1 | 1 | 1 | 1 | 1 | 1 | 0 | 0 | 1 | 1 | 1 | 1 | 1 | 1 | 1 |
| Mixed farm | 0 | 1 | 0 | 0 | 1 | 1 | 1 | 0 | 0 | 0 | 0 | 0 | 0 | 0 | 0 | 0 | 1 | 1 | 0 | 0 | 0 | 0 | 0 | 0 | 0 |
| Company Farm | 0 | 0 | 0 | 0 | 0 | 0 | 0 | 0 | 0 | 0 | 0 | 1 | 0 | 0 | 0 | 0 | 0 | 0 | 0 | 0 | 0 | 0 | 0 | 0 | 0 |
| Family farm | 1 | 1 | 1 | 1 | 1 | 1 | 1 | 1 | 1 | 1 | 1 | 0 | 1 | 1 | 1 | 1 | 1 | 1 | 1 | 1 | 1 | 1 | 1 | 1 | 1 |
| No loan farm | 1 | 0 | 0 | 0 | 0 | 0 | 0 | 0 | 0 | 1 | 1 | 1 | 0 | 0 | 0 | 0 | 1 | 1 | 0 | 0 | 1 | 0 | 1 | 1 | 1 |
| Loan Farm | 0 | 1 | 1 | 1 | 1 | 1 | 1 | 1 | 1 | 0 | 0 | 0 | 1 | 1 | 1 | 1 | 0 | 0 | 1 | 1 | 0 | 1 | 0 | 0 | 0 |
| Fertile soil | 0 | 0 | 0 | 0 | 0 | 0 | 0 | 0 | 0 | 1 | 1 | 1 | 0 | 0 | 0 | 1 | 1 | 1 | 0 | 1 | 1 | 1 | 0 | 0 | 1 |
| Less fertile soil | 1 | 1 | 1 | 1 | 1 | 1 | 1 | 1 | 1 | 0 | 0 | 0 | 1 | 1 | 1 | 0 | 0 | 0 | 1 | 0 | 0 | 0 | 1 | 1 | 0 |
| Soil health | 1 | 1 | 1 | 1 | 0 | 1 | 0 | 1 | 1 | 0 | 1 | 1 | 1 | 1 | 1 | 1 | 1 | 1 | 1 | 1 | 1 | 1 | 1 | 1 | 1 |
| Production potential | 1 | 1 | 1 | 1 | 1 | 1 | 1 | 1 | 1 | 1 | 1 | 1 | 1 | 1 | 1 | 1 | 1 | 1 | 1 | 1 | 1 | 1 | 1 | 1 | 1 |
| SCM practices | 1 | 1 | 1 | 1 | 1 | 1 | 1 | 1 | 1 | 1 | 1 | 1 | 1 | 1 | 1 | 0 | 1 | 1 | 1 | 1 | 1 |  | 1 | 1 | 1 |
| Climate | 1 | 1 | 1 | 1 | 1 | 1 | 1 | 1 | 1 | 1 | 1 | 1 | 1 | 1 | 1 | 1 | 1 | 1 | 1 | 1 | 1 | 1 | 1 | 1 | 1 |
| Change of income | 1 | 1 | 1 | 1 | 1 | 1 | 1 | 1 | 0 | 0 | 1 | 1 | 1 | 1 | 1 | 1 | 1 | 1 | 1 | 1 | 1 | 0 | 0 | 1 | 1 |
| Agri-environmental benefits | 1 | 1 | 1 | 1 | 1 | 1 | 1 | 1 | 1 | 1 | 1 | 1 | 1 | 1 | 1 | 1 | 0 | 1 | 1 | 1 | 1 | 1 | 0 | 1 | 1 |
| SCM cost | 1 | 1 | 1 | 1 | 1 | 1 | 1 | 1 | 1 | 1 | 1 | 1 | 1 | 1 | 1 | 1 | 1 | 1 | 1 | 1 | 1 | 1 | 1 | 1 | 1 |
| Govt org | 0 | 0 | 0 | 1 | 1 | 1 | 0 | 1 | 0 | 0 | 1 | 1 | 0 | 0 | 0 | 0 | 0 | 0 | 0 | 0 | 1 | 0 | 1 | 0 | 0 |
| Non Govt org | 1 | 1 | 1 | 1 | 1 | 1 | 1 | 1 | 1 | 1 | 1 | 1 | 0 | 1 | 1 | 1 | 1 | 1 | 1 | 1 | 1 | 1 | 1 | 0 | 0 |
| Farm research grant | 1 | 1 | 1 | 1 | 1 | 1 | 1 | 0 | 0 | 0 | 0 | 0 | 1 | 1 | 1 | 0 | 1 | 0 | 1 | 1 | 0 | 0 | 1 | 0 | 0 |
| Scientific support | 1 | 1 | 1 | 1 | 1 | 1 | 1 | 1 | 1 | 1 | 1 | 1 | 1 | 1 | 1 | 1 | 1 | 1 | 1 | 1 | 1 | 1 | 1 | 1 | 1 |
| Govt investments | 1 | 1 | 1 | 0 | 0 | 1 | 0 | 0 | 0 | 0 | 0 | 0 | 0 | 0 | 0 | 0 | 0 | 0 | 0 | 0 | 1 | 0 | 1 | 0 | 0 |
| Private investments | 0 | 0 | 0 | 0 | 0 | 1 | 1 | 1 | 1 | 1 | 1 | 1 | 0 | 0 | 0 | 0 | 1 | 0 | 0 | 0 | 1 | 0 | 1 | 0 | 0 |
| Carbon pricing and monitoring | 1 | 1 | 1 | 1 | 1 | 1 | 0 | 1 | 1 | 0 | 0 | 0 | 0 | 1 | 0 | 0 | 0 | 0 | 0 | 1 | 0 | 0 | 0 | 1 | 1 |
| Certainty of payment | 0 | 1 | 0 | 1 | 1 | 1 | 0 | 1 | 1 | 0 | 1 | 1 | 1 | 1 | 1 | 1 | 1 | 0 | 0 | 1 | 0 | 0 | 0 | 0 | 1 |
| Training and education supports | 1 | 1 | 1 | 1 | 1 | 1 | 1 | 1 | 1 | 1 | 1 | 1 | 1 | 1 | 1 | 1 | 1 | 1 | 1 | 1 | 1 | 1 | 0 | 1 | 1 |
| Expert information | 1 | 1 | 1 | 1 | 1 | 1 | 1 | 1 | 1 | 1 | 1 | 1 | 1 | 1 | 1 | 1 | 0 | 1 | 1 | 1 | 1 | 1 | 1 | 1 | 1 |
| Soil carbon policy | 0 | 1 | 1 | 1 | 1 | 1 | 0 | 0 | 0 | 0 | 0 | 0 | 1 | 1 | 0 | 0 | 1 | 1 | 1 | 1 | 1 | 0 | 0 | 1 | 1 |
| Social network | 0 | 1 | 1 | 1 | 1 | 1 | 1 | 1 | 1 | 1 | 1 | 1 | 1 | 1 | 1 | 0 | 1 | 1 | 1 | 1 | 0 | 1 | 1 | 1 | 1 |
| Trusted experts network | 1 | 1 | 1 | 1 | 1 | 1 | 0 | 1 | 1 | 1 | 1 | 1 | 1 | 1 | 1 | 1 | 1 | 1 | 1 | 1 | 0 | 0 | 0 | 1 | 1 |
| Govt officer | 0 | 0 | 0 | 0 | 1 | 0 | 0 | 1 | 1 | 0 | 0 | 0 | 0 | 0 | 0 | 0 | 0 | 0 | 0 | 0 | 0 | 0 | 0 | 0 | 0 |
| Independent advisors | 1 | 1 | 1 | 1 | 1 | 1 | 1 | 1 | 1 | 1 | 1 | 1 | 1 | 1 | 1 | 1 | 0 | 1 | 1 | 1 | 1 | 1 | 1 | 1 | 1 |
| Farmers | 1 | 1 | 1 | 1 | 1 | 1 | 1 | 1 | 1 | 1 | 1 | 1 | 1 | 1 | 1 | 1 | 1 | 1 | 1 | 1 | 1 | 1 | 1 | 1 | 1 |
| Scientists | 1 | 1 | 1 | 1 | 1 | 1 | 1 | 1 | 1 | 1 | 1 | 1 | 1 | 1 | 1 | 0 | 1 | 1 | 1 | 1 | 1 | 0 | 1 | 1 | 1 |
| Education institute | 1 | 1 | 1 | 0 | 1 | 1 | 1 | 1 | 1 | 0 | 0 | 0 | 1 | 1 | 1 | 0 | 1 | 1 | 0 | 1 | 1 | 0 | 0 | 0 | 0 |
| SCM attitude | 1 | 0 | 1 | 1 | 1 | 1 | 1 | 1 | 1 | 0 | 1 | 1 | 1 | 1 | 0 | 1 | 1 | 1 | 1 | 1 | 1 | 0 | 1 | 0 | 1 |
| Soil stewardship ethics | 0 | 0 | 0 | 1 | 1 | 1 | 0 | 1 | 1 | 1 | 1 | 1 | 1 | 1 | 0 | 1 | 1 | 0 | 1 | 1 | 1 | 0 | 1 | 0 | 1 |
| Technologies available | 0 | 1 | 0 | 0 | 0 | 0 | 0 | 0 | 1 | 0 | 0 | 0 | 1 | 0 | 0 | 0 | 0 | 0 | 1 | 1 | 1 | 0 | 0 | 1 | 1 |
| Trust | 1 | 1 | 0 | 1 | 1 | 1 | 1 | 1 | 1 | 1 | 1 | 1 | 1 | 1 | 0 | 1 | 1 | 1 | 1 | 1 | 1 | 0 | 1 | 1 | 1 |
| pH level | 0 | 1 | 0 | 1 | 1 | 0 | 0 | 1 | 1 | 0 | 0 | 0 | 1 | 1 | 1 | 0 | 1 | 0 | 0 | 0 | 0 | 0 | 1 | 0 | 0 |
| Soil moisture | 1 | 1 | 1 | 1 | 1 | 1 | 1 | 1 | 1 | 1 | 1 | 1 | 1 | 1 | 1 | 1 | 1 | 1 | 1 | 1 | 1 | 1 | 1 | 1 | 1 |
| Soil structure | 1 | 1 | 1 | 1 | 1 | 1 | 1 | 1 | 1 | 0 | 1 | 1 | 1 | 1 | 1 | 1 | 1 | 1 | 1 | 1 | 0 | 1 | 1 | 1 | 1 |
| Soil biodiversity | 0 | 1 | 1 | 1 | 1 | 1 | 1 | 1 | 0 | 0 | 1 | 1 | 1 | 1 | 1 | 1 | 1 | 1 | 1 | 0 | 0 | 1 | 1 | 1 | 1 |
| Landscape aesthetics | 1 | 0 | 1 | 1 | 1 | 1 | 0 | 1 | 1 | 1 | 0 | 0 | 1 | 1 | 1 | 1 | 0 | 0 | 0 | 0 | 0 | 1 | 1 | 1 | 1 |
| Soil water holding capacity | 1 | 1 | 1 | 1 | 0 | 0 | 1 | 1 | 1 | 1 | 0 | 0 | 1 | 1 | 1 | 1 | 1 | 1 | 1 | 1 | 1 | 1 | 0 | 0 | 1 |
| Soil erosion | 1 | 1 | 1 | 1 | 1 | 0 | 1 | 1 | 1 | 1 | 1 | 1 | 1 | 0 | 0 | 1 | 1 | 1 | 0 | 1 | 0 | 1 | 0 | 0 | 1 |
| Soil nutrients | 1 | 1 | 1 | 0 | 1 | 0 | 1 | 1 | 1 | 1 | 1 | 1 | 1 | 0 | 1 | 0 | 1 | 1 | 1 | 1 | 0 | 0 | 0 | 0 | 1 |
| Soil carbon content | 1 | 1 | 1 | 0 | 0 | 0 | 1 | 1 | 1 | 1 | 1 | 1 | 1 | 1 | 1 | 1 | 1 | 1 | 0 | 0 | 1 | 1 | 1 | 1 | 1 |
| Mental health | 1 | 1 | 1 | 1 | 0 | 1 | 0 | 1 | 0 | 0 | 0 | 0 | 1 | 0 | 0 | 1 | 0 | 1 | 0 | 0 | 0 | 1 | 0 | 0 | 0 |
| Shelter for livestock | 1 | 1 | 1 | 1 | 1 | 1 | 0 | 1 | 1 | 1 | 1 | 1 | 0 | 0 | 0 | 1 | 1 | 1 | 1 | 0 | 0 | 1 | 1 | 1 | 0 |


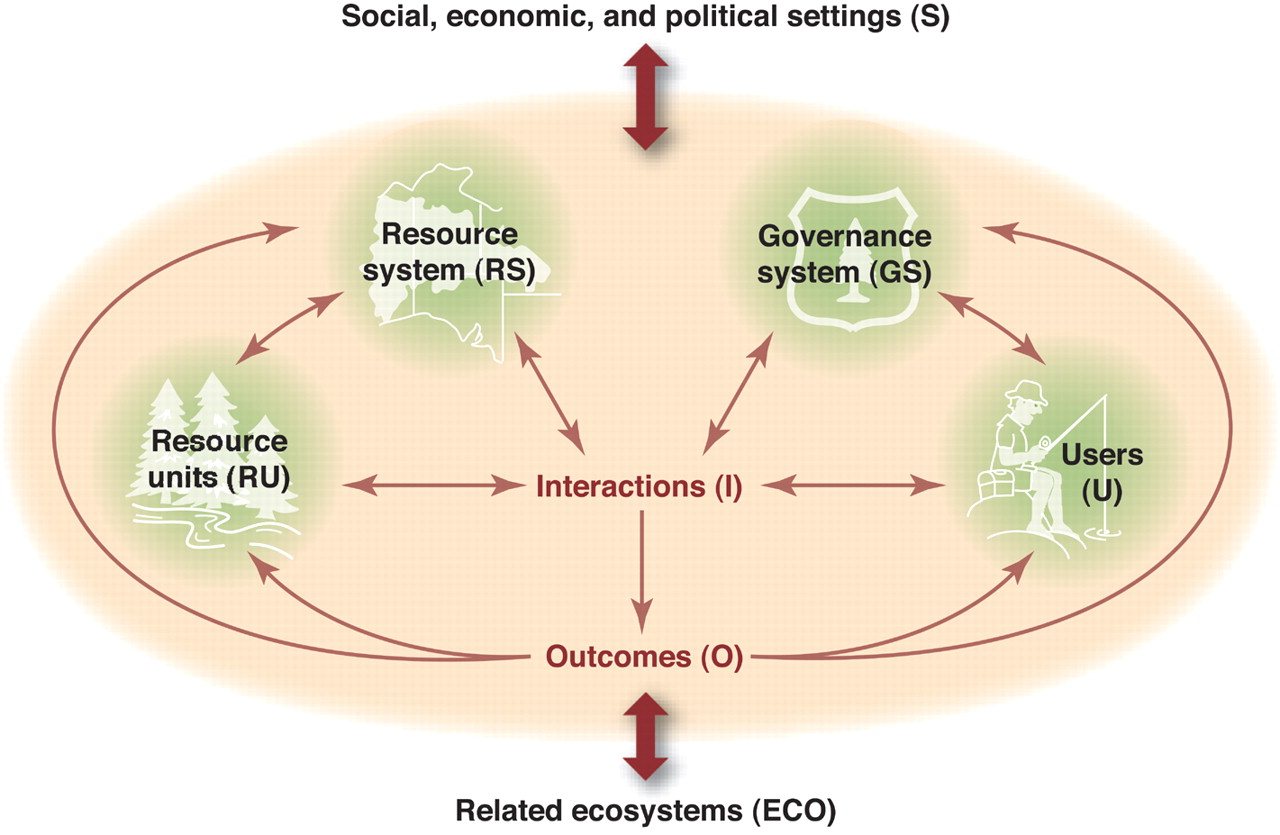


**SI Fig 1**. Ostrom’s social-ecological systems framework (Ostrom, 2009)


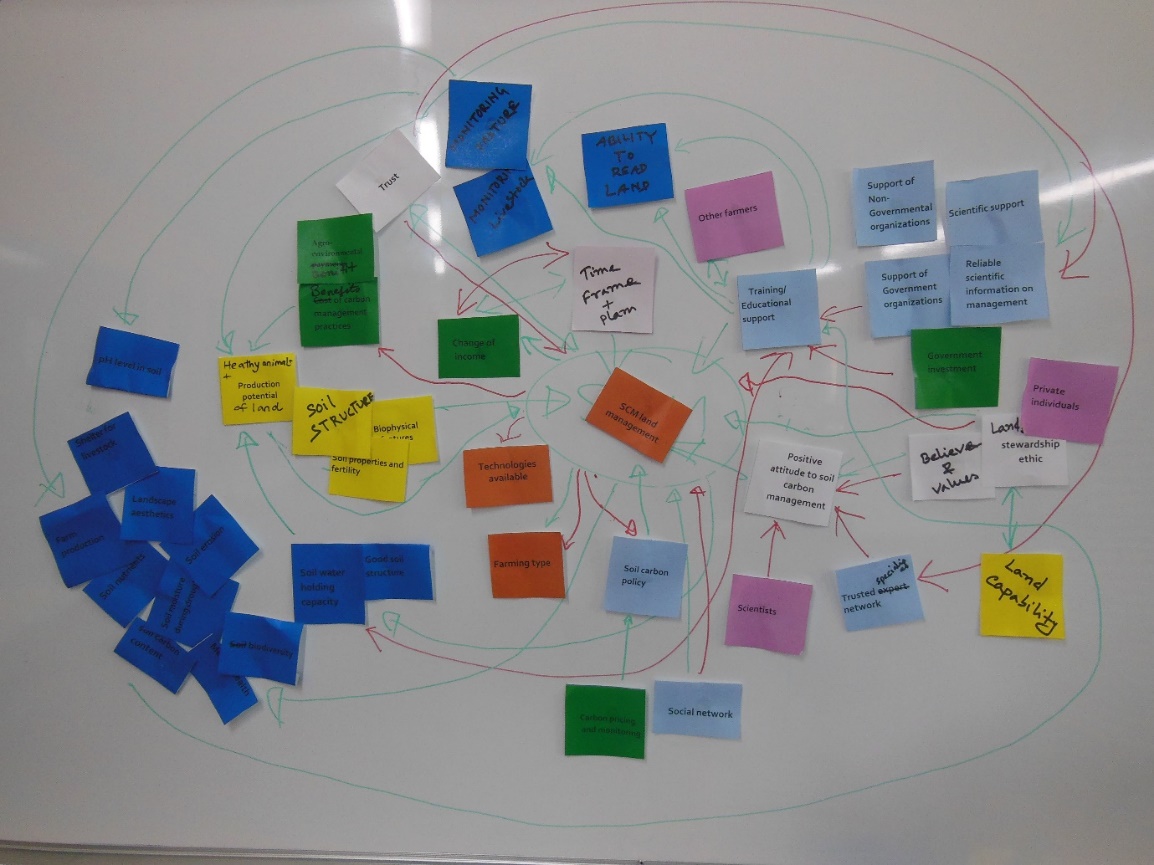


**SI Fig 2.** Causal loop diagram of farmers’ SES for SCM in rotational grazed land (first farmer workshop)


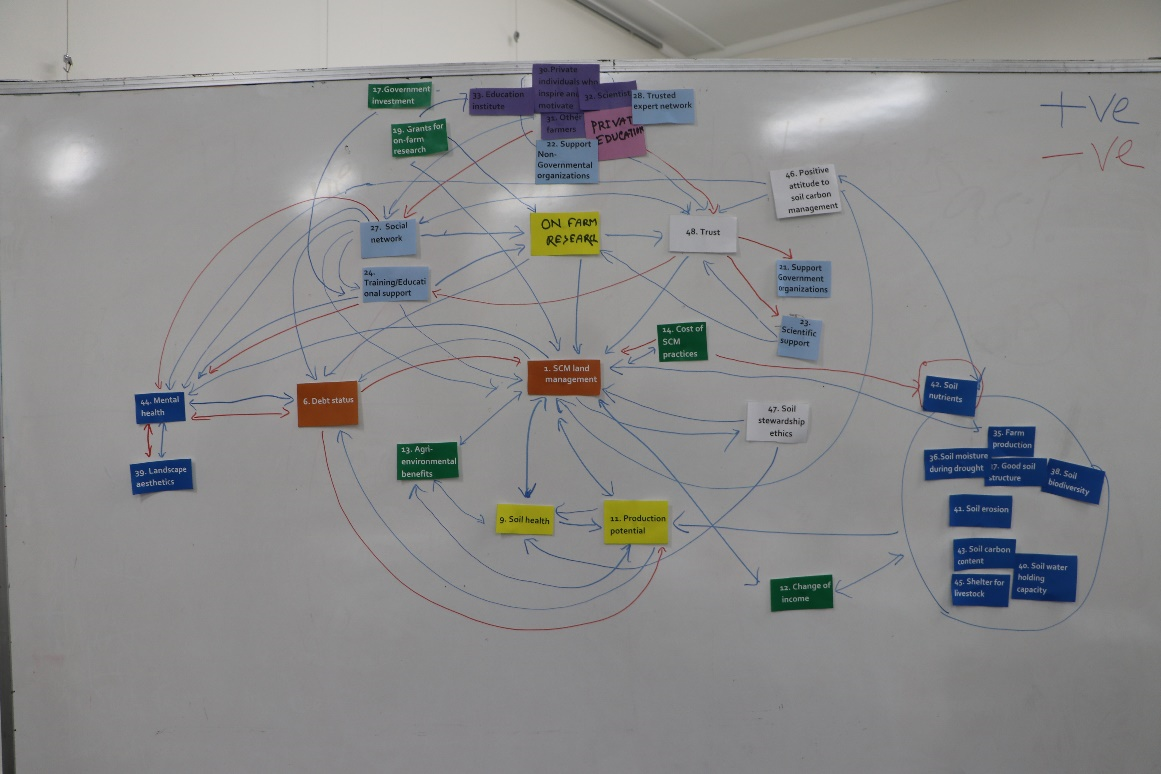


**SI Fig 3.** Causal loop diagram of farmers’ SES for SCM in rotational grazed land (second farmer workshop)


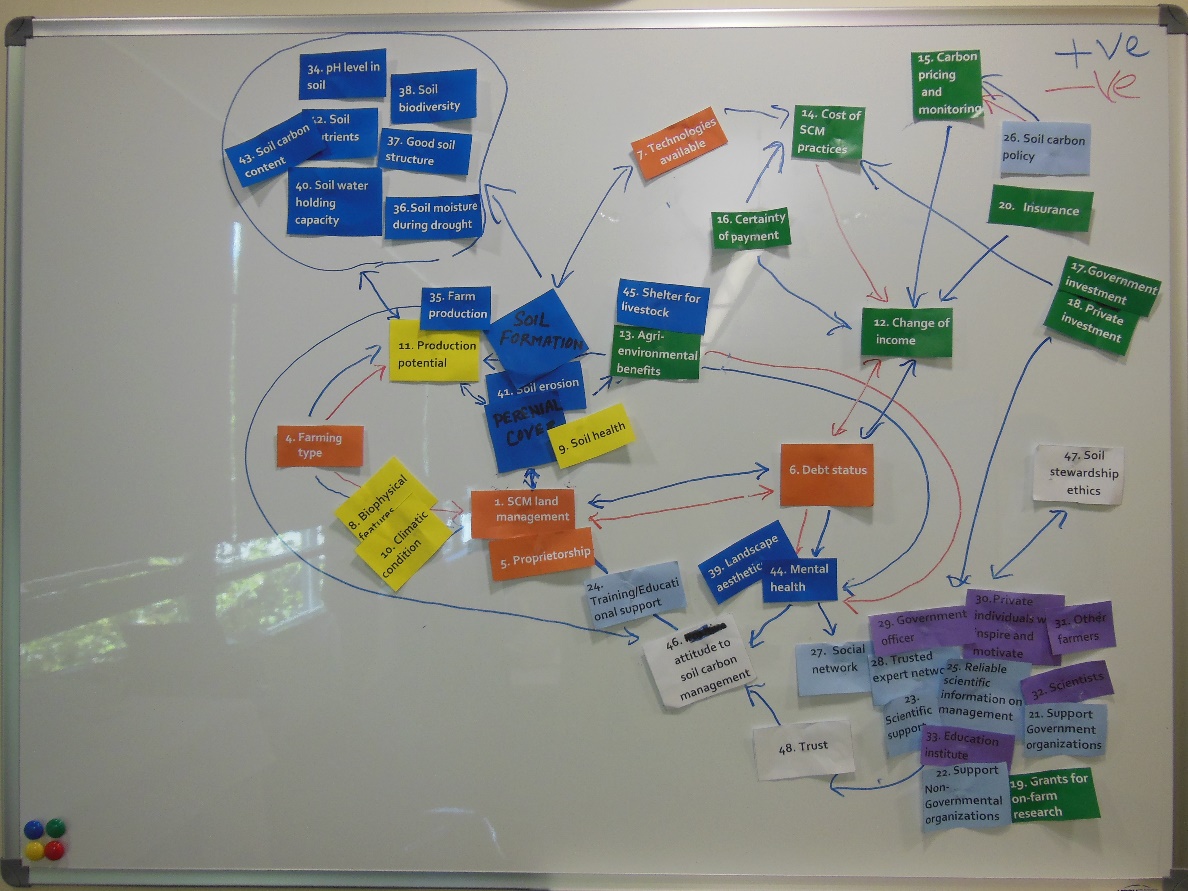


**SI Fig 4.** Causal loop diagram of farmers’ SES for SCM in rotational grazed land (first service provider workshop)


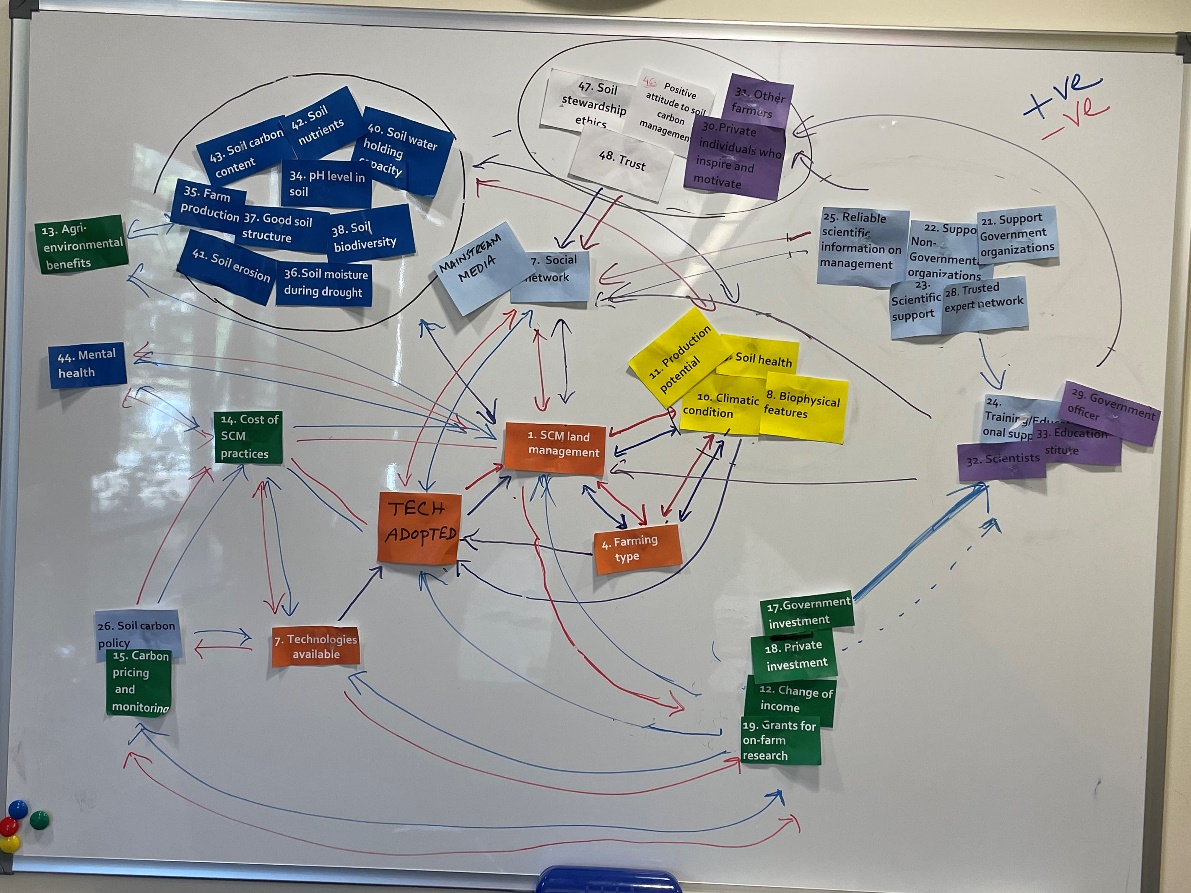


**SI Fig 5.** Causal loop diagram of farmers’ SES for SCM in rotational grazed land (second service provider workshop)

**Semi-structured Interview**

**Understanding the role of social-ecological system framework for examining farmers’ capacity to manage soil carbon**

Location………………………… Interview date………………….Farm number……………

**1. Demographics of interviewee**

**Q1.** What is your age?

**Q2.** Gender: Male Female Other Prefer not to say

**Q3.**Highest level of education reached:

**Q4.** Types of education: (a) General (b) Agriculture (c) Technical (d) Specialized (e.g. Master/PhD)

**Q5.** What is length of farming experience? (In years)

Q5b. What is the length of farming experience in current locality? (in years)

**Q6.** Do you have any off-farm income? (Y/N)

**2. Location Description**

**Q7**. What is the ownership status of your farmland?

1. Freehold
2. Company
3. Leased
4. Individual
5. family
6. Others (Specify)----------

**Q8.** What is the existing ownership status of your farm?
 (a) No loan

1. Easily manageable loan
2. Manageable loan but affect farm activity
3. Heavy commitments to loan
4. Other (Specify)

**Q9.** How many properties do you manage?

**Q10.**What is the total area of your farms? (ha)

**Q11.** What is your farm type?

1. Cropping
2. Grazing
3. Mixed (e.g. Cropping and Grazing)

**Q12**. What are the main type of soils on your farm? (Ask them to describe)

**Q13.** What is location of your farm/s? (Postcode)

**Q14.** Please describe your farming system and what would be the key reason you practice this form of farming? (Describe in your own words)

**3. Land management and factors affecting change**

**Q15**. What is your key goal for land management in the next five years?

**Q16.** What would be the main reason you would choose to change your land management?

**Q17.** Have you introduced or practice any of the land management practices at your farm*? (Tick all those that apply)*

| For cropping only farmers | | For mixed crop-livestock farmers/ Graziers | |
| --- | --- | --- | --- |
| No-till cropping practices |  | No-till cropping practices |  |
| Bio-char application |  | Bio-char application |  |
| Mulching bare soil |  | Mulching bare soil |  |
| Native vegetation area establishment |  | Increase area for pasture by decreasing area for crop |  |
| Inter cropping with perennial pasture |  | Inter cropping with perennial pasture |  |
| Perennial pasture |  | Perennial pasture |  |
| Tree planting |  | Tree planting |  |
| Planting tree belt for harvesting |  | Rotational grazing implementation |  |
| Stubble retention after crop harvest |  | Stubble retention after crop harvest |  |
| Use of compost |  | Legume in pasture |  |
| Others (specify) |  | Others (specify) |  |

**Q18.** What land management practice has the greatest potential for storing soil organic matter (SOM)? (Choose the best one according to your farm experience from question 21)

**Q19.** What other benefits would the practices you have chosen have for your land management?

**Q20.** Do you know the soil carbon/soil organic matter content of your farm soil before introducing that/those land management techniques?

**Q21.** Do you know the soil carbon / soil organic matter content at your farm after introducing that/those land management techniques?

**Q22.** When and how did you introduce the land management practices on your farm?

1. As part of own research/experiments
2. External interventions through project
3. Usual practice from before
4. Information from scientific sources
5. Information from trusted networks
6. Others (Specify)

**Q23.** Did you receive any financial support for changing your land management? If you have received funding can you explain in what form this is and how recent?

**Q24.** Have you received any training for making a change in land management practice? What forms of training have you received for making a change in land management practice?

1. On job training
2. Training from other farmers
3. Public meeting
4. Short course
5. None

**Q25.** What is the main constraint for managing your land?

**Q26.** Did the constraint alter with the change in land management?

**Q27a.** When you made a change in land management practice how did it affect your farm income?

1. Increased farm income in the short term
2. Reduced farm income in the short term
3. Increased farm income over the long term
4. Reduced farm income over the long term
5. No change in farm income

**Q27b.** What were the reasons for making a change in land management practice?

**Q28.** Was there any additional cost of implementing the land management on your farm? Can you estimate what that would be?

**Q29**. Was the physical environment (slope, location) a factor in making a change in land management practice/s? How does it influence?

**Q30**. What influence does climatic (e.g. temperature and rainfall variation) variables have in making a change in land management practice/s?

**Q31.** What soil properties were considered making a change in land management practice or practices? (Prompt if necessary (e.g. sand, silt, clay, and pH)

**Q32.**Would soil carbon pricing stimulate you to consider making a change in land management practice?

1. Assurance of return
2. Estimating the initial investment capacity
3. Clear cost and benefits estimates
4. Assumption of the capacity and environmental responsibility
5. Others (Specify)

**4. Role of public or private organisations or individuals in supporting change**

**Q33.** With making a change in land management practice was there any influence by government in that decision? If yes, what type of agency or individual on behalf of an organisation (public or private) was influential?

**Q34.** What are the types of public or private organisation initiative or program related to land management have you participated in? (Please name them and the time you were involved from)

**Q35**. Why did you participate in public or private organisation initiative or program related to land management?

**Q36**. Who supported your decision to change land management?

**Q37.** What are the types of organisations (private or public) or individuals were involved when you changed land management practices?

**Q38.** What were the main support from these organisations (private or public) or individuals in making a change in land management?

**Q39.** Can you tell me what you know of the government’s carbon policy and how your change in land management may be affected by it?

**Q40.** Do you know if your change in land management would be eligible for any government financial incentive or not?

**Q41.** Do you have any insurance cover for the farm? If yes what are the main insurance types needed for the farm?

1. Crop loss insurance
2. Livestock loss insurance
3. Income insurance
4. Insurance related to crop and livestock production
5. Others (specify)

**Q42**. What are the types of farmer social networks do you belong to?

__________________________________________________________

**Q43.** How long have you been part of the group? _________________(yrs)

**Q44.** Why are you participating in this network for soil management on your farm?

**Q45.** What is the main interest or focus of the group/s you participate in?

**5. Social networks for changing land management**

**Q46.** What are the types of people or groups you are involved with in relation to your farm business? Who is the most important organisation or person (public or private) for advice and guidance on land management especially when changing land management? (Pick one)

1. Local information groups
2. Extension worker (e.g. Government worker)
3. Local land services office (LLS)
4. Department of primary industry
5. Federal Department of Agriculture and Food
6. CSIRO
7. Government officials
8. Researcher
9. Office of Environment and Heritage (OEH)
10. Other ____________________________________

**Q47.** In your experience what is the most important reason for people or groups you nominated to successfully make a change in land management?

**Q48.** In your experience what are the main function of organisations operating in land management at farm level?

**Q49.** In your experience have you met any social barriers to changing land management practices where you live? In your experience how do social values and cultural norms influence soil management at the farm level?

**Q50.** What social and cultural factors influence farmers when they seek to change their land management to include carbon management?

**6. Feedback from making a change in land management**

**Q51.** Impact of changing your land management practices:

**Soil carbon:** (a) Increase (b) Decrease (c) No change (d) Don’t know

**pH:** (a) Increase (b) Decrease (c) No change (d) Don’t know

**Fertilizer application:** (a) Increase (b) Decrease (c) No change (d) Don’t know

**Soil moisture:** (a) Increase (b) Decrease (c) No change (d) Don’t know

**Facility of shelter for livestock:** (a) Increase (b) Decrease (c) No change (d) Don’t know

**Soil biodiversity:** (a) Increase (b) Decrease (c) No change (d) Don’t know

**Soil structure:** (a) Improved (b) Declined (c) No change (d) Don’t know

**Farm Production:** (a) Improved (b) Declined (c) No change (d) Don’t know

**Q52.** What would you suggest could strengthen the land management information sources and communication on carbon management in your area?

Thank you for your time and careful consideration of these questions…….. Would you be available for a workshop early next year to review the preliminary data?

**References**

Dumbrell NP, Kragt ME, Gibson FL (2016) What carbon farming activities are farmers likely to adopt? A best–worst scaling survey. Land Use Policy 54, 29-37.

Ostrom E (2009) A general framework for analyzing sustainability of social-ecological systems. science 325, 419-422.
